# Identification and Characterization of Copper-Responsive miRNAs and Their Target Genes in Jerusalem Artichoke

**DOI:** 10.3390/plants14060955

**Published:** 2025-03-18

**Authors:** Xi Chen, Tianyun Shao, Wenhan Dong, Jiayan Lin, Lixiang Dai, Yilong Ma, Zhaosheng Zhou, Xiaohua Long

**Affiliations:** 1 Jiangsu Provincial Key Laboratory of Coastal Saline Soil Resources Utilization and Ecological Conservation, College of Resources and Environmental Sciences, Nanjing Agricultural University, Nanjing 211800, China; 2 Institute of Crop Sciences, Inner Mongolia Academy of Agricultural & Animal Husbandry Sciences, Hohhot 010031, China

**Keywords:** miRNA, copper stress, Jerusalem artichoke (*Helianthus tuberosus*), target genes, qRT-PCR

## Abstract

microRNAs (miRNAs) are key regulators of gene expression in plants, significantly contributing to various biological processes and stress responses. While their roles have been extensively studied in *Arabidopsis thaliana* and other model plants, the response of miRNAs to copper (Cu) stress in Jerusalem artichoke remains unknown. This study addresses this gap by investigating Cu-responsive miRNAs and their regulatory roles in Jerusalem artichoke under Cu stress. Through small RNA library analysis, six miRNA families—miR168, miR394, miR397, miR398, miR408, and miR858—were identified in Cu-stressed and control plants of the Jerusalem artichoke cv. NY1. These miRNAs possess characteristic stem-loop precursor structures and detectable miRNA* sequences, with miR858 having unusually long precursors (1524–6448 nt). This study outlines a framework for miRNA-mediated Cu stress responses in Jerusalem artichoke, highlighting the roles of both well-established Cu-responsive miRNAs (miR397, miR398, and miR408) and other conserved miRNAs (miR168, miR394, and miR858). These miRNAs are suggested to influence Cu stress adaptation by modulating target genes involved in essential metabolic, physiological, and morphological processes, offering new insights into miRNA-mediated stress regulation in plants.

## 1. Introduction

Copper (Cu) is one of the eight essential micronutrients required for plant growth, playing crucial roles in enzyme catalysis, photosynthetic and respiratory electron transport, protein and carbohydrate metabolism, oxidative stress protection, cell wall strengthening, pollen formation, and seed production [1]. Cu deficiency in plants leads to various abnormal phenotypes, including structural changes in roots, stems, and leaves, significant chlorosis, poor pollen development, and reduced seed yield [2]. Conversely, excessive Cu can be toxic, causing rapid accumulation of reactive oxygen species, nutrient imbalances, and altered enzyme functions. This impairment affects photosynthesis by disrupting Photosystem II and severely reduces plant growth and yield [2,3]. In addition, excessive copper can also affect the absorption and utilization of other elements by plants. For example, the absorption of Fe, Zn, Ca, and Mn by the roots of sorghum decreases significantly as the Cu concentration increases, thus affecting the growth and development of the plants themselves [4]. Over the past few decades, excessive Cu stress has emerged as a significant environmental issue due to mining, sewage irrigation, and the use of Cu-containing pesticides, severely affecting plant growth and raising global concerns [5].

To mitigate Cu stress, plants have evolved a complex homeostatic network that regulates metal absorption, accumulation, transportation, and detoxification through physiological, biochemical, and molecular mechanisms [6,7]. Cu stress can activate a comprehensive range of plant defense mechanisms, such as the activation of efflux mechanisms and the induction of chelating proteins. Notably, plant hormones like abscisic acid (ABA), auxin (IAA), and ethylene (ET) play crucial roles in resisting Cu stress [8]. Studies have shown that ABA regulates the absorption and translocation of toxic metals by controlling stomatal closure [9]. Additionally, specific transcription factor families, particularly the large MYB family, play crucial roles in plant responses to heavy metals by binding to genes involved in metal binding, transport, and tolerance [10]. For instance, OsMYB84 positively regulates the Cu transport genes *OsCOPT2* and *OsHMA5*, modulating Cu uptake and translocation in rice (*Oryza sativa* L.) [11]. Certain MYB members in hot pepper (*Capsicum annuum* L.) are involved in responses to multiple heavy metals, including Cu [12]. Additionally, overexpression of RsMYB1 enhances anthocyanin accumulation in *Raphanus sativus* and increases Cu stress tolerance [13].

microRNAs (miRNAs) are endogenous, non-coding, single-stranded RNA molecules, typically 19–24 nucleotides (nt) in length, found in eukaryotes and some DNA viruses [14]. In plants, miRNAs are generated through transcription, precursor processing, and methylation, and subsequently guide the RNA-induced silencing complex (RISC) to target complementary mRNAs, regulating gene expression by cleaving mRNAs or inhibiting translation [15]. miRNAs play important roles in regulating plant growth, development, and stress responses [14]. Recent studies have gradually revealed the involvement of miRNAs in regulating both Cu deficiency and excess. In *Arabidopsis*, excess Cu reduces miR398 expression, leading to increased levels of *CSD1* and *CSD2* transcripts and proteins, while Cu deficiency raises miR398b and miR398c levels, resulting in decreased *CSD1* and *CSD2* transcripts [16,17]. Similarly, miR397, miR408, and miR857 play crucial roles in maintaining Cu homeostasis. Under low Cu availability, miR397 accumulates in plants such as grapevine and *Arabidopsis*, where it suppresses the synthesis of laccase and plastocyanin, facilitating Cu accumulation to restore homeostasis [18,19]. In *Arabidopsis*, miR408 and miR857 are also upregulated under Cu deficiency, targeting *LAC3/12/13* and *LAC7*, respectively, to downregulate their expression [18,20]. Simultaneous suppression of the conserved miR397, miR398, and miR408 families impairs photosynthesis and growth in low-Cu conditions, likely due to defects in plastocyanin biosynthesis [21].

Jerusalem artichoke (*Helianthus tuberosus* L.) is a perennial herb from the Asteraceae family, closely related to sunflowers (*Helianthus annuus* L.), and originates from eastern North America, but has since spread to Europe and Asia [22]. The tubers are utilized in food production, biomass feedstock, and the production of inulin and bioethanol, while its leaves are valuable for silage [23,24]. Jerusalem artichoke is well suited to grow in infertile, desert, and saline–alkali soils, highlighting its potential for both agricultural and environmental applications [25]. Furthermore, it demonstrates tolerance to heavy metals, with the NY5 variety showing promise for phytoremediation of cadmium-contaminated soils [26]. Although recent studies on *Helianthus tuberosus* miRNAs have primarily focused on families such as miR390 and miR396, systematic research on miRNAs responding to Cu stress remains lacking [27,28]. This study identifies and analyzes six miRNA families—miR168, miR394, miR397, miR398, miR408, and miR858—and their target genes associated with Cu stress response in *Helianthus tuberosus*. The findings will provide valuable insights into miRNA-mediated responses to Cu stress in *Helianthus tuberosus* and may inform future efforts to develop Cu-tolerant varieties.

## 2. Results

### 2.1. Analysis of Small RNA Sequencing Data

To investigate the roles of miRNAs in Jerusalem artichoke under Cu stress, two small RNA libraries were constructed: one from Cu-stressed leaves and one from control leaves. High-throughput sequencing yielded 26,560,000 raw reads for the Cu-stressed samples and 26,720,000 for the control samples. After filtering out low-quality reads, short reads (<15 nt), and reads detected only once in a single library, a total of 22,600,007 clean reads (85.09%) and 20,505,864 clean reads (76.74%) were obtained, corresponding to 2,852,207 and 2,157,954 unique reads for the Cu-stressed and control samples, respectively.

Next, the common and unique sequences between the two libraries were summarized. As shown in Figure S1, the two libraries shared 1,077,983 unique reads (27.41%), corresponding to a total of 35,674,780 reads (82.76%, with 17,839,090 from the control and 17,835,690 from the Cu-stressed library). The control-specific unique sequences numbered 1,079,971 (27.47%), corresponding to a total of 2,666,774 reads (6.19%), while the Cu stress-specific unique sequences totaled 1,774,224 (45.12%), corresponding to 4,764,317 total reads (11.05%) (Figure S1). Although the number of specific unique reads was high, their average detection frequency was relatively low, at 2.47 and 2.69 times, respectively. In contrast, the shared unique reads, though fewer in number, exhibited a much higher and more stable detection frequency, averaging 16.55 times.

**Figure 1:**
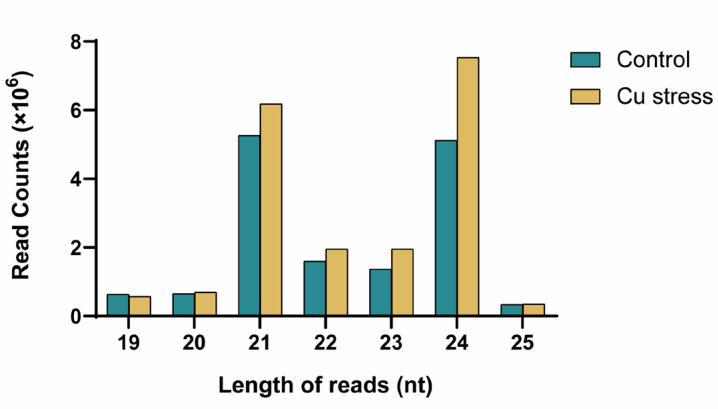
Size distribution of the total reads in the two libraries from *Helianthus tuberosus*.

The length distribution of reads in both libraries ranged from 15 to 44 nt, with the majority of sequences falling within the 19–25 nt range (Figure 1, Table S1). These sequences accounted for 91.48% and 95.03% of unique reads, and 73.05% and 85.20% of total reads, respectively. As shown in Table S1, 24 nt reads dominated among unique reads, comprising over half of the sequences, at 55.08% for the control and 56.21% for the Cu stress samples. The proportions of 21 nt and 23 nt reads were relatively close, ranging from 11.27% to 15.05%. For total small RNAs, 21 nt and 24 nt sequences were the most prevalent. In the control library, their proportions were similar, at 25.66% and 24.94%, while in the Cu stress library, they were higher at 27.35% and 33.36% (Figure 1; Table S1). The predominance of 24 nt small RNAs aligns with common sizes reported in other plants [29,30]. Notably, the proportion of 21 nt and 24 nt small RNAs was higher in the Cu stress library (60.71%) compared to the control (50.60%), suggesting a more extensive regulation of gene expression by small RNAs under Cu stress.

The classification of reads is crucial for identifying miRNAs. To further explore their functional composition, the reads were mapped to annotated tRNA, rRNA, and mRNA in the Jerusalem artichoke genome. This mapping revealed that 13.35% of unique reads and 48.15% of total reads in the control library, and 9.52% of unique reads and 32.07% of total reads in the Cu stress library, corresponded to these annotations. The annotation for snRNA and snoRNA was notably low, with 0.14% and 0.10% of unique reads in the control and Cu stress libraries, respectively, and total reads of 0.18% and 0.14%, respectively. As shown in Figure S2, the analysis revealed clear differences in small RNA composition between the two libraries. The control library had higher proportions of unique and total reads associated with snRNA, snoRNA, tRNA, rRNA, and mRNA compared to the Cu stress library. In contrast, the proportion of reads categorized as “others” was lower in the control library than in the Cu-stressed library.

### 2.2. Identification and Characterization of Differentially Expressed miRNAs in Two Libraries

**Figure 2:**
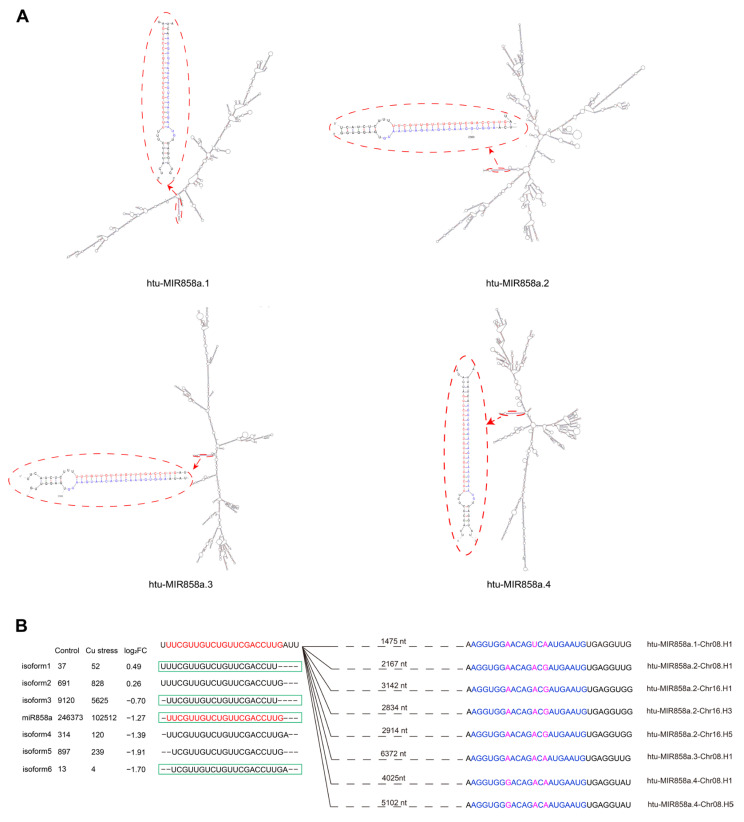
Predicted stem-loop structures of MIR858 (**A**) and isoforms aligning with MIR858 (**B**). The miR858 precursors were folded using RNA Folding Form V2.3, with sequences trimmed as indicated (see Table S3). Isoform sequences and their abundance were derived from control and copper stress libraries of *Helianthus tuberosus*. Red bases indicate the guide strand of miR858, while blue bases represent the passenger strand; purple fonts highlight base differences. The green rectangular box highlights the unique miR858 sequences found in other plant species (see Figure S4).

The “other reads” were mapped to the Jerusalem artichoke cv. NY1 transcripts assembled from our laboratory’s transcriptome sequencing and screened for miRNAs significantly altered under Cu stress based on miRNA identification criteria [31]. This process identified six miRNA families: miR168, miR394, miR397, miR398, miR408, and miR858 (Table 1). The miRNA precursors generally formed typical stem-loop structures (Figure 2 and Figure S3), with both the miRNA and miRNA* located on the stem and displaying minimal mismatches. Notably, all miRNA* sequences were detected in the two sRNA libraries, except for miR408b*, miR858a.1*, and miR858a.3*, which were found in other sRNA libraries from our laboratory (Table S2). Cloning verification was performed on the precursors and mature sequences of the six miRNA families in Jerusalem artichoke cv. NY1 (Table S3), excluding only the miR858 precursors.

Multiple sequence alignments of miR168, miR394, miR397, miR398, and miR408 family members from *Arabidopsis thaliana*, *Oryza sativa*, *Helianthus annuus*, and *Helianthus tuberosus* showed only 1–3 base differences or complete identity among members of the same miRNA family (Figure S4A). This finding indicates that these miRNAs are highly conserved across both dicotyledonous and monocotyledonous plants. Within the miR168 family, three precursor transcripts, named MIR168a.1, MIR168a.2, and MIR168b, were identified, producing two mature miRNAs: miR168a and miR168b. The precursor sequences range from 68 to 130 nt. miR168a and miR168a.1* exhibit three mismatches, while miR168a and miR168a.2* show two, and miR168b and miR168b* display one (Figure S3). Alignments revealed that the sequences of htu-miR168s are identical to those of han-miR168s and ath-miR168s, with slight variations from osa-miR168s. In the miR394 family, two precursor transcripts, MIR394a.1 and MIR394a.2, correspond to mature miR394a, both exhibiting three mismatches with their respective miRNA* sequences. However, the precursor lengths differ significantly, measuring 83 nt and 178 nt, leading to substantial differences in their hairpin structures (Figure S3). The sequence of htu-miR394a is completely identical across the four plant species, demonstrating its high level of conservation.

In the miR397 family, a single hairpin-forming transcript, named MIR397a, was identified, producing mature miR397a. This miRNA has two mismatches with miR397a*, and the precursor is 67 nt long (Figure S3). htu-miR397a differs by only one nucleotide from osa-miR397b and ath-miR397b, while being identical to osa-miR397a, ath-miR397a, and han-miR397a (Figure S4). Within the miR398 family, three precursor transcripts—MIR398a, MIR398b, and MIR398c—were identified, each producing a mature miRNA (miR398a, miR398b, and miR398c). The precursor sequences of MIR398a and MIR398b are highly similar, both being 61 nt long, with only one base difference between the mature miR398a and miR398b, resulting in similar hairpin structures (Figures S3 and S5). In contrast, miR398c has three mismatches with miR398c*, and its precursor is 148 nt long, with a distinct hairpin structure compared to MIR398a and MIR398b. htu-miR398a matches han-miR398a, htu-miR398b matches osa-miR398b, and htu-miR398c matches ath-miR398a-3p, han-miR398b, and osa-miR398a.

In the miR408 family, three hairpin-forming precursor transcripts, MIR408a, MIR408b, and MIR408c, were identified, corresponding to mature miRNAs miR408a, miR408b, and miR408c. These precursor sequences are highly similar, with only 3 to 5 nt differences among them, resulting in very similar hairpin structures (Figures S3 and S5). All three miRNAs exhibit four mismatches with their respective miRNA* sequences, and the precursor lengths range from 81 to 83 nt. In the other three species, we were unable to identify sequences that perfectly matched htu-miR408a, htu-miR408b, and htu-miR408c, as these sequences exhibited at least one nucleotide difference. Nonetheless, we detected a 21 nt sequence in the library that was identical to osa-miR408a-3p, as well as another 21 nt sequence that matched han-miR408a exactly. The most abundant sequences were the 20 nt long htu-miR408a and htu-miR408b.

Regarding miR858, four distinct precursors were identified on the transcript: MIR858a.1, MIR858a.2, MIR858a.3, and MIR858a.4, with precursor lengths of 1524 nt, 2959 nt, 6448 nt, and 5223 nt, respectively (Table S3). These precursors form highly complex hairpin structures and correspond to the same mature miR858a, with mismatch numbers between miR858a and miR858a.1*, miR858a.2*, miR858a.3*, and miR858a.4* being two, one, one, and one, respectively (Figure 2A). A total of 65 miR858 sequences were examined from miRBase, PmiREN, and the literature, revealing only 1–5 base differences among them (Figure S4, Table S4) [32]. Among these, 22 sequences were identical to htu-miR858a, and 7 sequences representing three isoforms were detected in the *Helianthus tuberosus* sRNA libraries. Additionally, four unique isoforms of htu-miR858a were identified in the same libraries, showing slight 1–2 base shifts either upstream or downstream of miR858a (Figure 2B).

**Figure 3:**
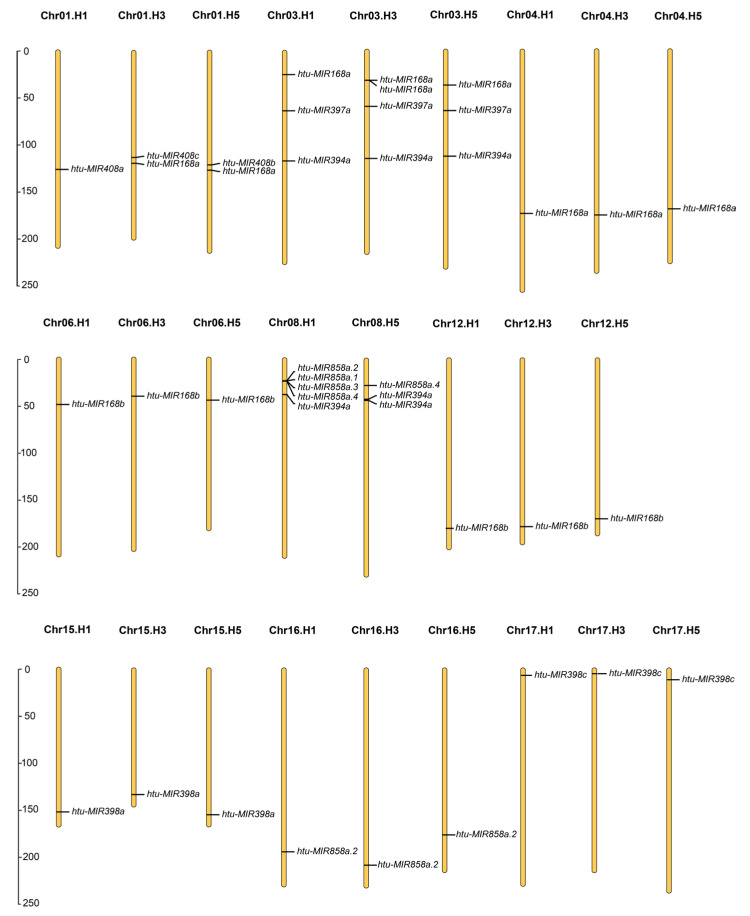
Genomic localization of the six Cu-responsive miRNA families in *Helianthus tuberosus*. Numerical labels above chromosomes indicate their numbers. The units on the left are megabases (Mb).

The miRNAs from the Jerusalem artichoke cv. NY1 were analyzed using the newly published genome sequence [33], revealing a comprehensive genome consisting of 17 homologous groups and six pseudochromosomes per group. Using stringent criteria, 41 miRNA precursors were identified from this reference genome (Figure 3, Table S3). Notably, the precursor of miR398b was mapped exclusively to Chr03.H1, Chr03.H2, and Chr15.H1 in the complete genome sequence (version1) (Table S3) [33]. The sequences derived from genomic alignment, when compared with the cloned sequences, showed only minor differences in base pairs, with most nucleotides being identical. The miR168 family had the highest number of precursors, totaling 15, followed by miR398, miR858, and miR394, with 9, 8, and 6 precursors, respectively. The miR397 and miR408 families each had three precursors, which were insufficient to construct a phylogenetic tree. The localization of *MIRNAs* in the reference genome was complex, with most genes scattered across multiple chromosomes (Figure 3). The 41 *MIRNA* genes were distributed across 26 chromosomes. Five genes were located on Chr08.H1 (*htu-MIR858as*, and *htu-MIR394a*), and four genes were found on Chr03.H3 (*htu-MIR168as*, *htu-MIR394a*, and *htu-MIR397a*). Three genes were located on each of Chr03.H1, Chr03.H5, and Chr08.H5, while two genes were present on Chr01.H3 and Chr01.H5. The remaining chromosomes each contained one gene: Chr01.H1, Chr04.H1/H3/H5, Chr06.H1/H3/H5, Chr12.H1/H3/H5, Chr15.H1/H3/H5, Chr16.H1/H3/H5, and Chr17.H1/H3/H5. The phylogenetic trees of the miR168, miR394, miR398, and miR858 families are displayed in Figure S6. For miRNAs that can be localized to different positions, those located on the same chromosome group demonstrate a higher degree of sequence similarity, implying closer evolutionary relationships. For instance, the four *MIR168a.2s* on Chr03, the three *MIR168bs* on Chr06, the other three *MIR168bs* on Chr12, and the three *MIR168a.1s* on Chr04 exhibit strong genetic similarity. In contrast, *MIRNAs* from different chromosome groups exhibit more distant evolutionary relationships, reflecting the complex evolution and distribution of miRNAs.

To investigate the transcriptional regulatory mechanisms of miRNA and their roles in the regulatory network, the 2000-base pair upstream region of each *MIRNA* was extracted for *cis*-acting element prediction analysis. In addition to core promoter elements such as the TATA-box and the commonly found CAAT-box, 30 specific promoter *cis*-acting elements were identified (Table S5). These elements were classified into five categories: light-responsive, hormone-responsive, stress-responsive, biosynthesis- and metabolism-related elements, and other elements (such as A-box, 3-AF3 binding sites, 60K protein binding sites, Box II, Box III, high-transcription-level elements, HD-Zip 3, cell cycle regulation, and AT-rich sequences) (Figure S7). Among these, light-responsive elements were the most abundant, accounting for 44% of the *cis*-elements found in the 44 *MIRNA* genes. Hormone-responsive elements were the second most common (28.68%), with abscisic acid-responsive elements comprising 8.90% and present in the promoters of 39 *MIRNA* genes. Stress-responsive elements (18.79%) were the third most common, including elements associated with anaerobic induction (126), anoxic response (15), low-temperature response (50), drought response (31), defense and stress response (26), and wound response (1). Other *cis*-acting elements identified were related to circadian control, flavonoid biosynthesis regulation, MYBv1 binding site, zein metabolism regulation, meristem expression, seed-specific regulation, and endosperm expression. Notably, the *MIR168a.2-Chr03.H5* promoter contained the highest number of *cis*-acting elements (69), followed by the *MIR168a.2-Chr03.H3 (1)* (54) and *htu-MIR168a.2-Chr03.H1* promoters (45), which had numerous elements that could regulate miRNA expression and enable broad functionality in Jerusalem artichoke (Table S5). These findings indicate that miRNAs respond to Cu stress while also playing essential roles in a broader regulatory network that manages various environmental stresses.

### 2.3. Identification and Characterization of miRNA Targets

**Figure 4:**
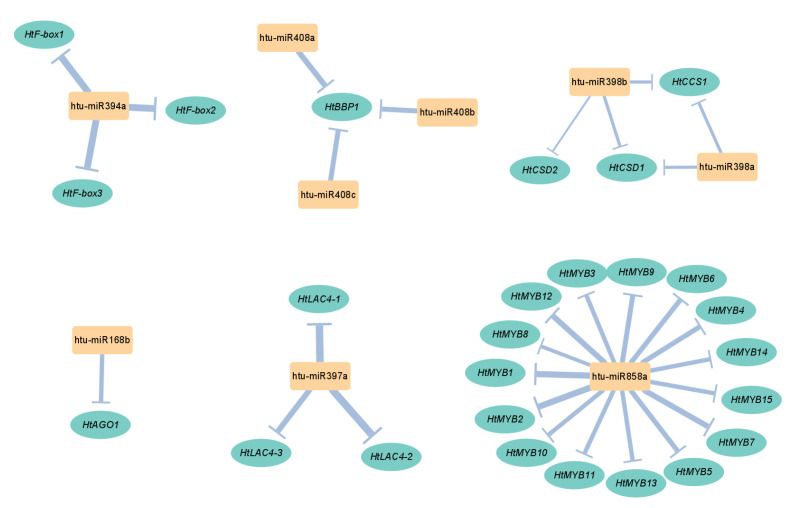
The specific profiles of Cu-responsive miRNA–target networks in *Helianthus tuberosus*. The nodes represent miRNAs (orange boxes) or target genes (green ovals). The line thickness indicates the number of mismatches between the miRNAs and their target genes, with thicker lines representing fewer mismatches and thinner lines indicating more.

miRNAs regulate gene expression by binding either perfectly or near perfectly to their target mRNAs, resulting in either target transcript cleavage or translational repression. Integration of transcriptome and degradome data identified 26 target genes from six miRNA families. These miRNA–target pairs include miR168-*AGO* (*Argonaute*), miR394-*F-box*, miR397-*LAC* (*Laccase*), miR398-*CCS* (*Copper chaperone for superoxide dismutase*)/*CSD* (*Copper/Zinc superoxide dismutase*), miR408-*BBP* (*Basic blue protein*), and miR858-*MYB* (*Myeloblastosis*) (Figure 4).

**Figure 5:**
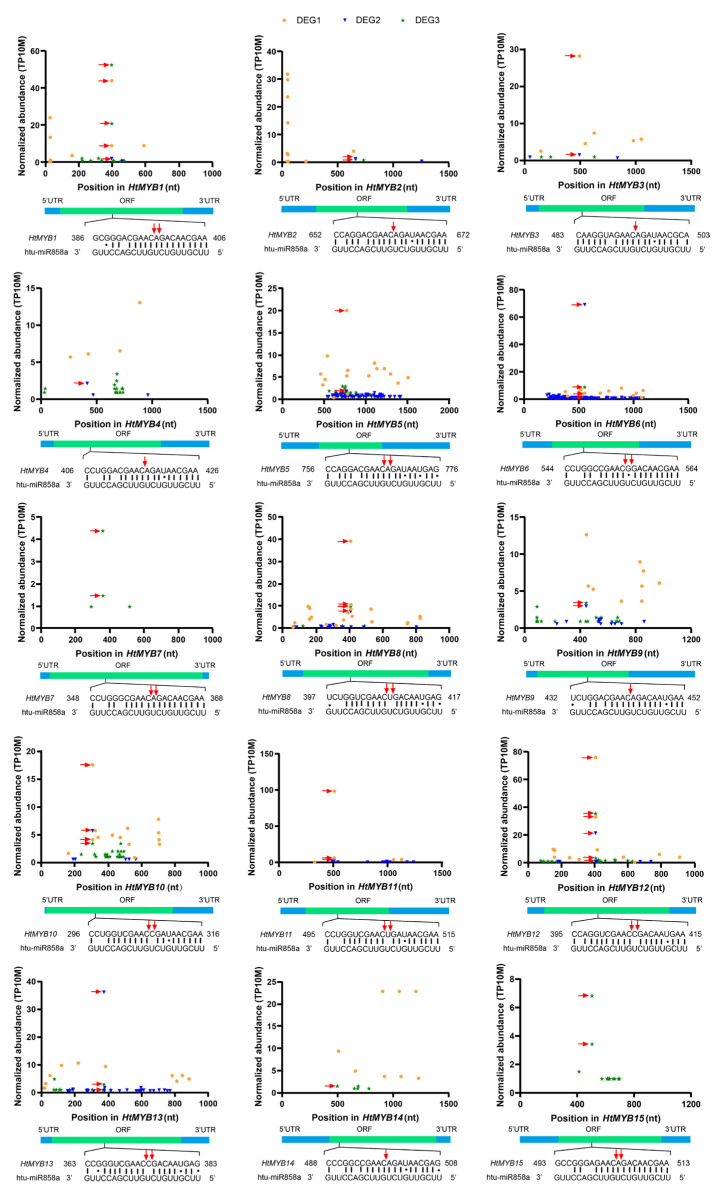
t-plots of miR858 targets identified from three degradome libraries derived from *Helianthus tuberosus*. Fifteen *HtMYB* genes were targeted by miR858 from degradome libraries of DEG1 (mixed roots and leaves of seedlings), DGE2 (tubers), and DEG3 (mature leaves during flowering). Degradome tag abundance is normalized to the total number of tags in the library per ten million (TP10M). The cleavage sites of genes targeted by miRNAs (9–11th, 5′→3′) and their corresponding degradome tag abundance are both marked with red arrows. The open reading frames (ORFs) and 5′ and 3′ untranslated regions (UTRs) of the targets are displayed with green and blue boxes, respectively. Aligned sequences are represented with vertical lines and dots indicating perfect pairing and G-U wobbles.

Three sets of degradome data from *Helianthus tuberosus* were used to construct t-plot graphs, selecting the miRNA with the fewest mismatches to its target gene as a representative (Figure 5 and Figure S8). For example, miR168a has two mismatched bases at the binding site with *HtAGO1* and shows two adjacent cleavage sites at positions 509–511. miR394a exhibits a single base mismatch at the seventh position of the binding site with *HtF-box1/2/3*. The *HtF-box1* gene has a cleavage site at positions 1435–1436, while *HtF-box2* and *HtF-box3* have two adjacent cleavage sites at positions 1348–1350 and 1240–1242, respectively. miR397a contains three base mismatches at the binding sites of *HtLAC4-1*, *HtLAC4-2*, and *HtLAC4-3*, each with a single cleavage site at positions 817–818, 1040–1041, and 729–730, respectively. miR398 targets the mRNAs of *HtCCS1*, *HtCSD1*, and *HtCSD2*, with 4–5 mismatches at their binding sites. Specifically, *HtCSD2* is targeted by miR398b, while *HtCCS1* and *HtCSD1* are targeted by all miR398 isoforms (a, b, and c). The *HtCCS1* gene exhibits three adjacent cleavage sites at positions 855–858, while *HtCSD1* and *HtCSD2* have two and one cleavage sites at positions 108–110 and 581–582, respectively. Finally, miR408 has three mismatches at the complementary site with *HtBBP1*, which features a cleavage site at positions 94–95.

**Figure 6:**
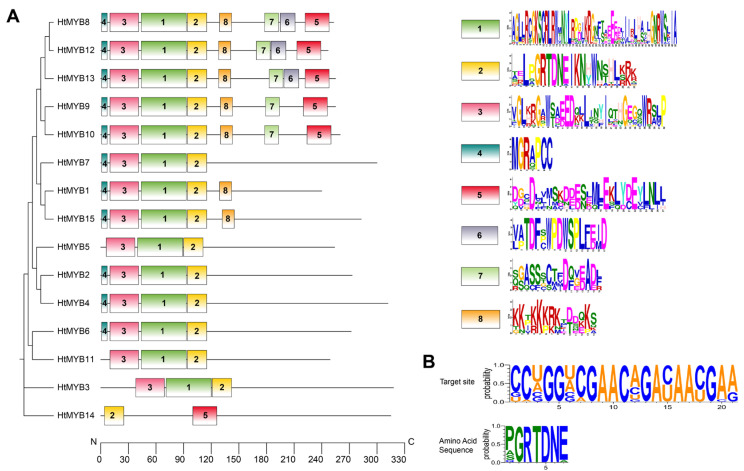
Conserved motifs in HtMYBs (**A**) and sequence features of *HtMYBs* targeted by miR858 (**B**). Shown in panel A, eight motifs, marked in different colors, were identified using the MEME tool. Sequence logos were generated with WebLogo (https://weblogo.threeplusone.com/create.cgi, revision 3), accessed on 10 July 2024, where the height of each letter represents the probability at each position. In panel B, the upper image shows the *HtMYB* sequences targeted by miR858 (1st–21st, 5′→3′), while the lower image displays the corresponding encoded amino acids.

In our investigation of miR858, we identified 15 target genes, designated as *HtMYB1* through *HtMYB15*. These genes show varying levels of mismatch with miR858: three mismatches (*HtMYB2*, *HtMYB3*, *HtMYB7*, and *HtMYB12*), four mismatches (*HtMYB1*, *HtMYB4*, *HtMYB6*, *HtMYB10*, and *HtMYB11*), five mismatches (*HtMYB5*, *HtMYB9*, *HtMYB13*, *HtMYB14*, and *HtMYB15*), and six mismatches (*HtMYB8*). Specific cleavage sites at positions 9 to 11 within the complementary bases between miR858 and various *HtMYB* genes were identified: *HtMYB2* at positions 662–663, *HtMYB3* at 493–494, *HtMYB4* at 416–417, *HtMYB9* at 442–443, and *HtMYB14* at 498–499. Additionally, *HtMYB1*, along with *HtMYB5*, *HtMYB6*, *HtMYB7*, *HtMYB8*, *HtMYB10*, *HtMYB11*, *HtMYB12*, *HtMYB13*, and *HtMYB15*, exhibited two adjacent cleavage sites at positions 396–398, 766–768, 554–556, 358–360, 407–409, 306–308, 505–507, 405–407, 373–375, and 503–505 (Figure 5). Further alignment analysis of these *HtMYBs*, focusing on a 50-base region starting from the 9th base complementary to miR858, revealed that these regions are all different, indicating that miR858 mediates diverse mRNA degradation processes across various *HtMYB* genes (Figure S9). Despite this sequence diversity at the binding sites, all 15 *HtMYBs* deduce a conserved amino acid sequence encoding P/A/S/QGRTDNE (Figure 6B).

Among the 26 target genes identified from the six miRNA families, all but *HtCSD1*, which has its cleavage site located in the 5′ UTR, have their cleavage sites within the open reading frames (ORFs) of their respective genes. Based on the relative abundance of degradome tags at miRNA target sites, the targets were classified into three categories: Category I represents the most reliable targets, including genes with the highest tag abundance at positions 9–11; Category II consists of genes with tag abundance between the maximum and median; and Category III includes all remaining targets [34,35]. Among the identified targets, *HtLAC4-1/2* were classified into Category II, *HtCSD2* into Category III, and the remaining 23 targets into Category I.

Subsequently, bioinformatics analyses were conducted on the 26 target genes identified from the transcripts (submitted to GenBank: PQ368255–PQ368280). First, alignment of these target genes with the annotated reference genome revealed that each target gene corresponded to one to three highly matching annotated genes, and degradome analysis confirmed that these genes were cleaved by miRNAs (Table S6). Second, most genes within the same family exhibited notable consistency in several predicted biochemical properties, including CDS length, molecular weight, isoelectric point, and hydrophilicity. Specifically, the *HtAGO1* gene has a CDS length of 3177 bp, and the protein it encodes is localized in the nucleus with a predicted molecular weight of 117.49 kDa. The *HtF-box* genes targeted by miR394 have CDS lengths of approximately 1400 bp, and the proteins they encode have predicted molecular weights around 50 kDa and generally high isoelectric points near 9, although their subcellular localizations differ. The *HtLAC4* family genes exhibit high uniformity, with CDS lengths ranging from 1668 to 1689 bp. The deduced proteins have isoelectric points between 9.21 and 9.33, and all are localized to the chloroplast. The miR398 target genes, excluding *HtCCS1* (which has a slightly longer CDS and higher molecular weight), exhibit similar properties to *HtCSD1* and *HtCSD2*, including isoelectric points ranging from 5.61 to 6.33 and moderate hydrophobicity. In contrast, the *HtBBP1* gene has a notably shorter CDS of 381 bp and encodes a protein with a high isoelectric point of 9.68.

The HtMYB family, which was a focus of this study, comprises genes with CDS lengths ranging from 723 bp (*HtMYB1*) to 957 bp (*HtMYB3*). The deduced proteins have amino acid lengths ranging from 240 (HtMYB1) to 318 (HtMYB3) residues, with molecular weights varying from 27.22 kDa (HtMYB1) to 163.63 kDa (HtMYB11). Their isoelectric points range from 4.84 (HtMYB4) to 6.20 (HtMYB6), except for HtMYB5, which exhibits an alkaline property with a pI of 8.96. Online predictions of subcellular localization indicated that all proteins are localized to the nucleus, with GRAVY values ranging from −0.912 (HtMYB5) to 0.832 (HtMYB8). As shown in Figure 6A, conservation analysis revealed that motif 2 is present in every HtMYB, underscoring its fundamental role in the biological function of HtMYBs. Figure 6B further illustrates that the amino acids encoded by regions complementary to miR858a within the 15 target genes constitute part of motif 2. Additionally, motifs 1 and 3 are consistently observed in all HtMYB members except HtMYB15. The degree of similarity in these motifs correlates with the phylogenetic relatedness of the proteins, suggesting that variations in these motifs may confer distinct functional capabilities. Notably, HtMYB14 diverges from other MYB proteins. Our structural analysis of the genomic sequence for HtMYB14 identified motifs 3 and 4; however, an extended transcript sequence providing a comprehensive representation of HtMYB14 was not found within the assembled transcripts, indicating a potential gap in the current transcriptome assembly.

### 2.4. Expression Levels of miRNAs and Target Genes Across Different Tissues

**Figure 7:**
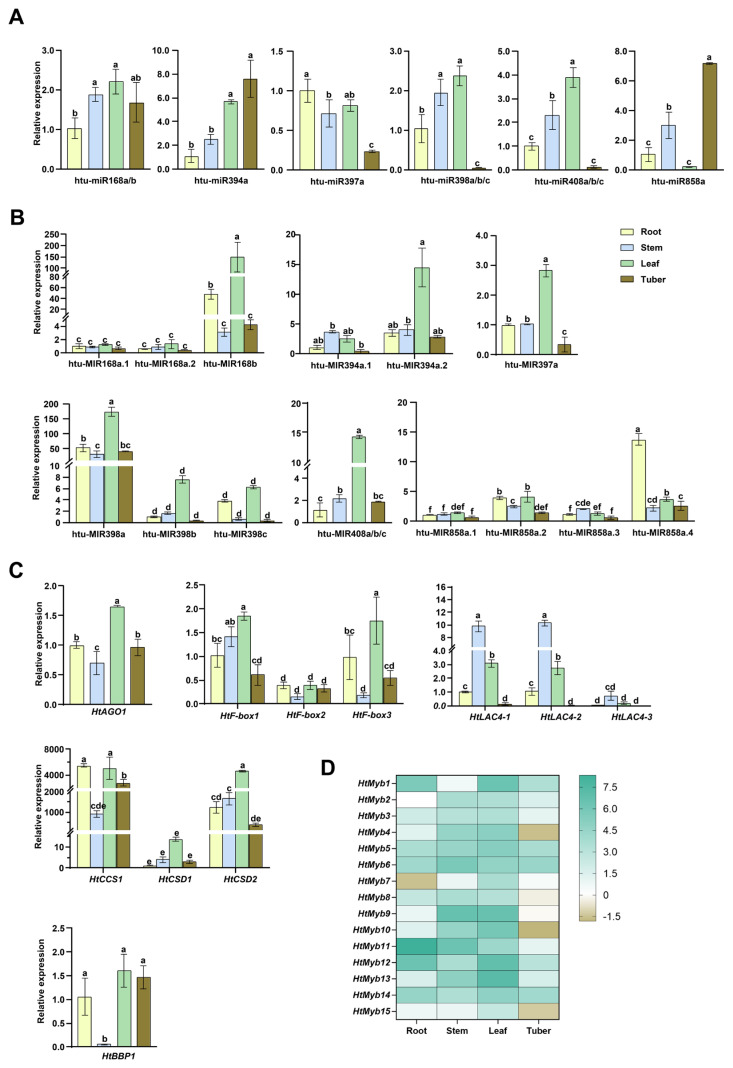
Tissue expression analysis of Cu-responsive miRNAs (**A**), MIRNAs (**B**), and their targets (**C**,**D**) in *Helianthus tuberosus* by qRT-PCR. qRT-PCR experiments used the value of the first gene in the roots as the reference (set to 1) for each section, except for *MIR398b*, *HtCDS1*, and *HtMyb2*, where root values were also set to 1. *HtU6* snRNA served as a reference for miRNAs, while *HtActin* was used for the normalization of both MIRNAs and target genes. Bar graphs representing relative expression levels were calculated using the 2^−ΔΔCT^ method. Differences in expression levels were assessed using one-way ANOVA followed by Duncan’s post hoc test. Values are mean ± SD (n = 3), with lowercase letters indicating significant differences (*p* ≤ 0.05). The heatmap was generated using −ΔΔCT values, with green indicating high expression levels and brown indicating low expression levels.

The systematic organization and stage-specific expression of miRNAs provide insights into their molecular regulation throughout plant growth and development. This study analyzed the expression patterns of miRNA precursors, mature miRNAs, and their target genes in the young roots, stems, and leaves of 14-day-old seedlings, as well as in mature tubers of Jerusalem artichoke cv. NY1, using qRT-PCR. As shown in Figure 7, within the MIR168 family, MIR168b exhibited the highest expression in leaves, significantly surpassing MIR168a.1/a.2 across all tissues, with no significant differences observed among the latter. This pattern mirrors that of miR168a/b, with higher levels in leaves and stems compared to roots and tubers. The target gene *HtAGO1* also showed the highest expression in leaves, followed by roots and tubers, with the lowest expression in stems.

In the MIR394 family, MIR394a.2 is highly expressed in leaves, while both MIR394a.1 and MIR394a.2 show low expression in other tissues. miR394a expression is higher in leaves and tubers compared to roots and stems. Among the *HtF-box* genes targeted by miR394, *HtF-box2* exhibits low expression across all tissues, lower than that of *HtF-box1* and *HtF-box3*. Both *HtF-box1* and *HtF-box3* are highly expressed in leaves, followed by roots. *HtF-box1* shows the lowest expression in tubers, while *HtF-box3* has the lowest expression in stems, followed by tubers. Notably, all three *HtF-box* genes are expressed at very low levels in mature tubers, contrasting with the high expression of miR394 in tubers (Figure 7A–C).

MIR397a is expressed at twice the level in leaves compared to roots and stems, whereas miR397a expression is slightly lower in leaves than in roots. In tubers, MIR397a expression is less than half of that in roots and stems, with miR397a showing a similar pattern. The three *HtLAC4* genes targeted by miR397a exhibit consistent expression trends across tissues, with the highest levels in stems, followed by leaves, then roots, and the lowest in tubers. Compared to *HtLAC4-1* and *HtLAC4-2*, *HtLAC4-3* shows lower expression in roots, stems, and leaves. The lower abundance of *HtLAC4s* in roots contrasts with the high expression of miR397a in roots. In tubers, the low expression of *HtLAC4s*, miR397a, and MIR397a suggests that these genes may not play a significant role in tuber development (Figure 7A–C).

In the MIR398 family, MIR398a is expressed at significantly higher levels than MIR398b/c across all tissues, with MIR398a/b/c showing the highest expression in leaves and relatively lower levels in roots, stems, and tubers. Similarly, the expression profiles of miR398a/b/c are higher in leaves and stems compared to roots and tubers. Among the targets of miR398, *HtCCS1* and *HtCSD2* are highly expressed in all four tissues, especially compared to *HtCSD1*. Consistent with the expression patterns of the miR398 precursors and mature sequences, *HtCCS1*, *HtCSD1*, and *HtCSD2* all exhibit high expression in leaves (Figure 7A–C).

The precursors and mature sequences of miR408 exhibit consistent expression trends, with the highest levels in leaves, followed by stems, and then in roots and tubers. The target gene of miR408, *HtBBP1*, shows similar expression levels in roots, leaves, and tubers, all of which are higher than in stems (Figure 7A–C).

The MIR858 family exhibits a diverse expression pattern. Generally, MIR858a.4 shows the highest expression across tissues, followed by MIR858a.2, and then MIR858a.1 and MIR858a.3. MIR858a.4 is significantly more expressed in roots, followed by leaves, stems, and tubers. MIR858a.2 has similar expression levels in roots and leaves, higher than in stems and tubers. MIR858a.1 is most highly expressed in leaves compared to other tissues, while MIR858a.3 is more expressed in stems, followed by leaves, roots, and tubers. Unlike the precursors, mature miR858a is predominantly expressed in tubers, followed by stems, roots, and leaves (Figure 7A, B). The heatmap shows that the expression levels of the 15 *HtMYBs* targeted by miR858a vary significantly across tissues (Figure 7D). *HtMYB5/6/12/14* are relatively highly expressed, while *HtMYB2/3/7/8/15* show lower expression levels across tissues. *HtMYB4/9/10/13* are more highly expressed in stems and leaves but lower in roots and tubers. *HtMYB1* is more expressed in roots and leaves, followed by tubers and stems; *HtMYB11* shows higher expression in roots and stems, then in leaves, and lastly in tubers. Overall, these 15 targets display lower expression in mature tubers and higher in seedling leaves, which contrasts with the expression pattern of miR858a, underscoring the strong targeting relationship between miR858a and the *HtMYBs*, as well as their crucial role in seedling growth and tuber development.

### 2.5. Expression Analysis of miRNAs and Their Target Genes Under Cu Stress

**Figure 8:**
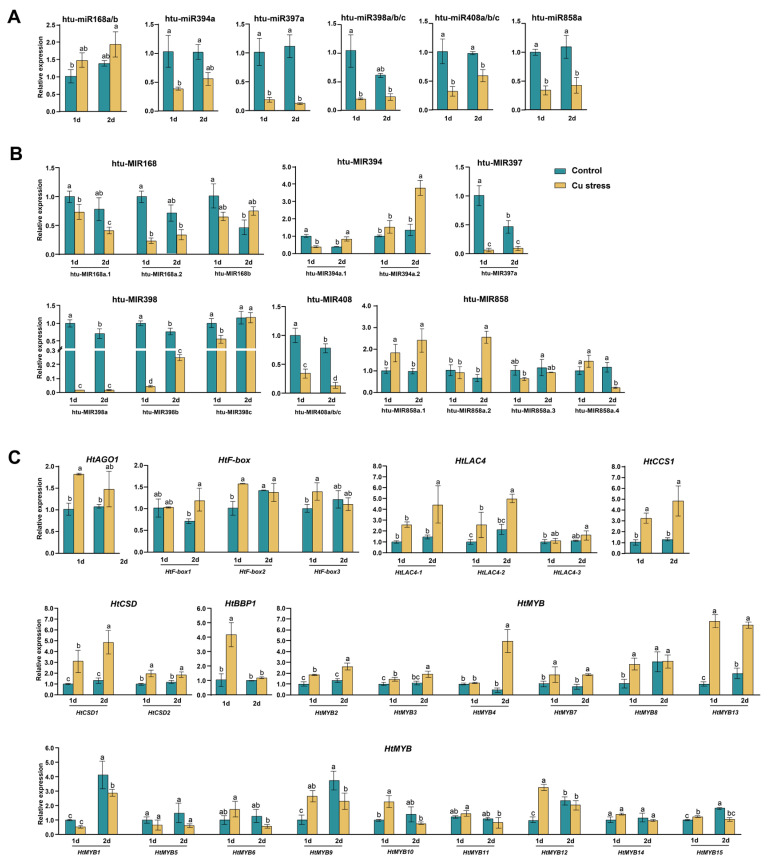
Expression analysis of Cu-responsive miRNAs (**A**), MIRNAs (**B**), and their targets (**C**) in *Helianthus tuberosus* under varying Cu stress durations. Seedlings hydroponically cultivated for 4 weeks were transferred to nutrient solutions with identical compositions, differing only in copper concentration: 0.15 μM (control) and 150 μM (treatment). Leaves were harvested after 1 and 2 days. qRT-PCR experiments were normalized to the value of each gene on the first day under control conditions, set to 1. Values are mean ± SD (n = 3), with lowercase letters indicating significant differences (*p* ≤ 0.05). Refer to Figure 7 for additional information.

The sRNAome analysis revealed that, after 2 days of excessive Cu stress, significant changes occurred in most members of the six miRNA families in the leaves of Jerusalem artichoke cv. NY1. Notably, miR168a and htu-miR398c showed only slight increases (Table 1). Among these, miR168b exhibited a significant increase, while miR394a, miR397a, miR398a/b, miR408a/b/c, and miR858a demonstrated notable decreases. The qRT-PCR analysis of mature miRNAs confirmed that these trends aligned with the sRNAome data, observed at both 1 and 2 days of Cu stress (Figure 8A).

We also conducted qRT-PCR analysis on the precursors and targets of these miRNAs after 1 and 2 days of Cu treatment (Figure 8B,C). In the miR168 family, both MIR168a.1 and MIR168a.2 showed declining trends, while MIR168b initially decreased before increasing. Its target gene, *HtAGO1*, also increased, reflecting trends observed in miR168a/b. For the miR394 family, MIR394a.1 initially decreased and then increased, whereas MIR394a.2 exhibited a gradual increase. The three target genes, *HtF-box1/2/3*, showed slight increases post-Cu stress, indicating an inverse relationship with miR394. In the miR397 family, MIR397a expression significantly decreased following Cu stress treatment, similar to miR397a. Conversely, the corresponding target genes *HtLAC4-1/2/3* increased after Cu stress. For the miR398 family, MIR398a/b showed a decline under Cu stress, consistent with miR398a/b expression, while MIR398c initially decreased and then returned to control levels. Conversely, target genes *HtCCS1*, *HtCSD1*, and *HtCSD2* were significantly upregulated. In the miR408 family, Cu stress led to downregulation of MIR408a/b/c, consistent with miR408a/b/c expression. The target gene *HtBBP1* significantly increased on the first day and showed a slight increase on the second day.

The four precursors of the miR858 family exhibited diverse expression patterns under Cu stress. MIR858a.1 and MIR858a.2 significantly increased after 2 days, while MIR858a.3 and MIR858a.4 decreased over the same period. Among the target genes of miR858a, *HtMYB2*, *HtMYB3*, *HtMYB4*, *HtMYB7*, and *HtMYB13* consistently increased after both 1 and 2 days of Cu stress, contrasting with the trend of miR858a. *HtMYB8* and *HtMYB12* showed notable increases on the first day but remained unchanged on the second day. *HtMYB9*, *HtMYB10*, and *HtMYB15* were significantly upregulated on the first day but exhibited decreases on the second day. *HtMYB1* and *HtMYB4* did not show significant changes on the first day; however, by the second day, *HtMYB4* increased significantly, while *HtMYB1* decreased (Figure 8B, C).

## 3. Discussion

Since the discovery of miRNAs in *Arabidopsis thaliana*, research on miRNAs and their targets has expanded significantly [36]. However, the study of miRNAs in Jerusalem artichoke remains relatively new. Early research included the prediction of 21 miRNAs using expressed sequence tags (ESTs) by Barozai et al. and Monavar Feshani et al. [37,38]. Later studies, such as Wen et al., examined the miR390-TAS3-ARF pathway under salt stress, and Ding et al. investigated the miR396-*HtGRFs*-*HtSCL33*-*HtWRKY40* pathway under heat (42 °C) and cold (4 °C) stresses [27,28]. In this study, we identified six miRNA families—miR168, miR394, miR397, miR398, miR408, and miR858—in Jerusalem artichoke cv. NY1 by analyzing two sRNA libraries. These miRNAs meet the criteria for miRNA identification, which include non-coding precursors forming hairpin structures and both miRNA and miRNA* being detectable on the stem-loop [31]. The identification of these miRNAs has significantly expanded the known miRNA repertoire in Jerusalem artichoke. Genomic localization and cloning confirmed these identifications.

Among the 16 precursors identified from the transcripts, except for miR858, their lengths ranged from 61 to 178 nucleotides, which is consistent with the typical hairpin size of 146 nucleotides observed in most known miRNAs [39]. However, miR858 precursors were unusually long, ranging from over 1524 to 6448 nucleotides. Although mature miR858 sequences have been identified in several plants, the full characterization of its precursor was only recently achieved due to its extraordinary length and structure. Wang et al. reported that miR858 precursors in seed plants can vary from 350 to 5500 nucleotides, far exceeding the typical length of miRNA hairpins previously observed in Jerusalem artichoke [27,28,32,37,38], highlighting a unique feature of miR858 in this species. Apart from the high sequence similarity among miR408a/b/c and miR398a/b precursors (Figure S5), the precursors of other miRNA families displayed significant variability (Figure S6, Table S3). Despite this variability, the mature sequences of miRNA families exhibited only 1–3 nucleotide differences within the family (Table 1), and 0–5 nucleotide differences compared to other species (Figure S4), indicating a high level of conservation within plant miRNA families. The nucleotide differences between miRNA precursors derived from the transcriptome and those from the genome (Table S3) are likely due to the different varieties of Jerusalem artichoke used in this study. The transcript-derived data were obtained from ‘Nanyu No. 1’, while the genomic data were sourced from ‘Yulin’. Furthermore, variations within the same chromosome group, such as miR408a/b/c on chromosome group 1 and miR398a/b on chromosome group 15, are likely attributed to Jerusalem artichoke’s hexaploid genome, which resulted from a hybridization event between tetraploid and diploid Helianthus species, followed by chromosome doubling [33].

In plants, miRNAs guide the RISC to target mRNAs, leading to post-transcriptional gene silencing through near-perfect base complementarity [14]. Through degradome analysis, 26 target genes from six miRNA families were identified in Jerusalem artichoke for the first time, including *HtAGO1*, *HtF-boxes*, *HtLAC4s*, *HtCCS1*, *HtCSDs*, *HtBBP1*, and *HtMYBs* (Figure 4). Among these targets, 23 (88.46%) were classified as Category I, 2 (7.69%) as Category II, and 1 (3.85%) as Category III (Figure 5 and Figure S8, Table S7), demonstrating the high reliability of these results.

At the post-transcriptional level, miRNAs regulate plant growth and development [14,40]. The identification of *cis*-acting elements related to light response and MeJA responsiveness in the promoter sequences of six miRNA families in Jerusalem artichoke supports their involvement in these processes (Table S5, Figure S7). Tissue-specific expression analysis revealed that, aside from *LAC4s* being most abundant in the stems, followed by leaves, miR168, miR397, miR398, and miR408, along with their targets, were all highly abundant in leaves, indicating their crucial role in plant growth. In contrast, miR394 and miR858 were highly expressed in mature tubers, while their target genes (*F-boxes* and *MYBs*) showed lower expression, suggesting that these miRNAs may negatively regulate tuber development (Figure 7). Moreover, discrepancies were observed between the expression patterns of certain miRNA precursors and their mature sequences, such as between miR394a and MIR394a.1/2, and between miR858a and MIR858a.1/a.2/a.3/a.4 (Figure 7). These inconsistencies suggest that precursor levels do not always correlate with mature miRNA levels, potentially due to the complex regulation of miRNA biogenesis and other factors affecting miRNA stability and RISC formation [41].

miRNAs play a critical role in regulating gene expression under various stress conditions, including heavy metal stress, which is vital for plant adaptation [42,43]. Excessive Cu disrupts plant physiological functions and poses environmental and health risks through soil contamination [2]. Recent studies underscore the importance of miRNAs in managing Cu stress responses, with validations in species such as *Arabidopsis thaliana*, *Paeonia ostii*, and *Vitis vinifera* [44,45,46,47]. Our study highlights miRNA and target gene responses in Jerusalem artichoke under Cu stress (Table 1, Figure 8). Conserved miRNAs such as miR397, miR398, and miR408 regulate copper-associated proteins including LACs, CCSs, CSDs, and BBPs under Cu stress. In Jerusalem artichoke, these miRNAs were significantly downregulated in response to Cu stress (Table 1, Figure 8), consistent with findings in other species [17,18,19,48], indicating a conserved regulatory mechanism across plants [16,49]. miR397 primarily targets *LACs*, influencing laccase activity and cell wall lignification, thereby affecting cell growth, reproductive organ development, and stress tolerance [50]. Additionally, miR397 targets *CKB3*, a key regulator of circadian rhythm and flowering in *Arabidopsis* [51], though these targets were not identified in our study. The downregulation of miR398 under Cu stress leads to the upregulation of *CCS* and *CSD*, which mitigate Cu toxicity and support plant growth [16]. Similarly, miR408 regulates *BBP* and other genes, enhancing photosynthesis, antioxidant capacity, and stress tolerance [52]. Under high-Cu conditions, the suppression of miR408 increases *BBP* expression, facilitating Cu uptake and reactive oxygen species (ROS) regulation [53]. Although miR857 was shown by Abdel-Ghany et al. to mediate Cu stress by suppressing *LAC7* expression in *Arabidopsis*, it was not detected in our study [18].

In addition to the well-known Cu-responsive miRNAs, miR168, miR394, and miR858 in Jerusalem artichoke have also been implicated in the Cu stress response (Table 1, Figure 8). miR168 targets AGO1, a protein crucial for recruiting miRNAs to the RISC, mediating mRNA cleavage or translational repression [54]. The CRISPR-Cas9 knockout of *OsMIR168a* in rice resulted in disrupted miRNA-mRNA pathways and altered phenotypes, such as accelerated seedling growth and early maturation [55]. Furthermore, the miR168-*AGO1* interaction has been linked to heavy metal stress responses, with reduced miR168 levels observed in grapevines under Cu stress and in rice under Cd stress [47,56]. In this study, increased miR168 expression under Cu stress was accompanied by elevated *AGO1* levels, suggesting enhanced miRNA regulation and potential disruptions in feedback mechanisms within the miRNA pathway. This phenomenon may be attributed to the presence of a long non-coding RNA (lncRNA) in Jerusalem artichoke that binds to miR168, buffering its inhibitory effect on AGO1 through a sponge-mediated mechanism [57]. miR394 and its target F-box proteins, which are highly conserved, are key components of the SKP1-Cullin/CDC53-F-box ubiquitin ligase complex involved in substrate degradation [58,59]. In *Arabidopsis*, upregulation of miR394 and downregulation of its target, *LEAF CURLING RESPONSIVENESS* (*LCR*), have been associated with increased tolerance to drought and cold, although this also raises sensitivity to salt stress [60]. Under Cd stress, miR394 expression is downregulated in *Brassica napus*, whereas it is induced in barley [61,62]. Our study found that miR394 expression was downregulated under Cu stress, with its target F-box proteins upregulated, suggesting a role in the plant’s Cu stress response (Figure 8). The precursors of miR858 are structurally distinct, while its mature sequences target MYB transcription factors and are conserved across plant species [32]. Overexpression of miR858 has been shown to increase anthocyanin content in *Malus spectabilis* and enhance flavonoid accumulation in lettuce [63,64,65]. In *Arabidopsis*, pri-miR858a encodes miPEP858a, a small peptide that regulates miR858a expression and influences plant growth and flavonoid levels [66]. Our study observed that miR858 interacts with 15 *MYBs* in response to Cu stress, with its downregulation derepressing these *MYBs* and potentially enhancing their transcription factor activity. This role of MYBs in the Cu stress response has also been noted in hot pepper and Petunia [12,13]. MYB transcription factors are involved in the regulation of key metal transporter genes like *HMA*, highlighting the complexity of the miR858-MYB regulatory module [67]. We speculate that the upregulation of certain MYB transcription factors in Jerusalem artichoke under Cu stress may enhance Cu tolerance; however, further validation using methods such as the yeast two-hybrid system is needed.

**Figure 9:**
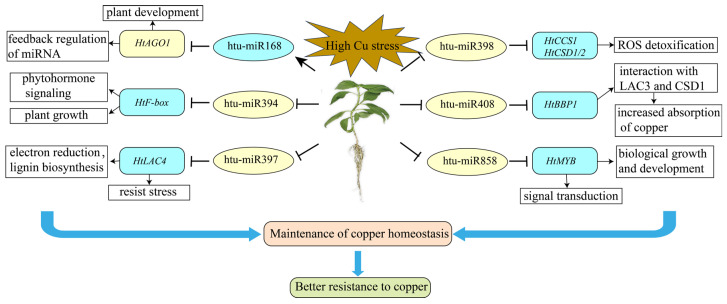
The potential regulatory network of Cu-responsive miRNAs in *Helianthus tuberosus*. The network illustrates changes in the expression profiles of miRNAs and their target transcripts identified in *Helianthus tuberosus* under copper stress, incorporating functional insights from previous studies. Blue shading indicates upregulation, while yellow shading indicates downregulation.

Ultimately, a model for the molecular mechanisms underlying Jerusalem artichoke’s response to Cu stress has been proposed (Figure 9). This model illustrates that both well- established Cu-responsive miRNAs (miR397, miR398, and miR408) and other miRNAs (miR168, miR394, and miR858) regulate metabolic, physiological, and morphological processes at the post-transcriptional level under excessive Cu stress.

## 4. Materials and Methods

### 4.1. Plant Culture and Treatment

Fresh tubers of Jerusalem artichoke (cv. Nanyu No.1, NY1) were collected from Xinyang Agricultural Experimental Station (33°32′12″ N, 120°26′22″ E), Yancheng City, Jiangsu Province, China. The tubers were thoroughly washed with deionized water, and the buds were sliced into approximately 10 mm × 10 mm × 3 mm pieces. These pieces were placed in an enamel tray containing sterilized quartz sand and moistened with deionized water. Initial cultivation was conducted in darkness at 22 °C using a plant incubator. After germination, the seedlings were transferred to a growth chamber (KeSheng, Ningbo, China) set to 25/18 °C (day/night) with a light intensity of 120 μmol m^−2^ s^−1^ and a 12 h photoperiod. They were irrigated with half-strength Hoagland solution (pH = 5.8). After 2 weeks, the seedlings were moved to pots with the same nutrient solution for an additional 2 weeks, with the solution refreshed every 3 days [27].

Uniform seedlings were then treated with either the half-strength Hoagland solution containing 0.15 μM CuCl₂ as the control or with an increased CuCl_2_ concentration of 150 μM to induce Cu stress. Roots, stems, and leaves were collected after 1 and 2 days of treatment, frozen in liquid nitrogen, and then stored at −80 °C until further analysis. Each treatment was replicated three times.

### 4.2. RNA Extraction and sRNA Library Construction and Sequencing

Total RNA was extracted from roots, stems, leaves, and tubers stored at −80 °C using Trizol reagent (Takara, 9109, Kusatsu, Japan). RNA quality was assessed with a NanoDrop 2000 spectrophotometer (Thermo Fisher Scientific, Waltham, MA, USA), and RNA integrity was confirmed by gel electrophoresis. For sRNA library construction, RNA extracted from leaves treated with 0.15 μM and 150 μM Cu for 2 days was used. The small RNA (sRNA) libraries were prepared and sequenced by BGI Tech (Shenzhen, China). The procedure included isolating 18–30 nt sRNA fragments using 15% denaturing polyacrylamide gel electrophoresis, sequential ligation of 3′ and 5′ adaptors to the sRNA molecules, cDNA synthesis, PCR amplification, selecting 110–130 base pairs (bp) fragments via 8% polyacrylamide gel electrophoresis, quality control, circularization, and then sequencing on a DNBSEQ-G50 platform.

### 4.3. sRNA-seq Data Processing and miRNA Identification

Raw sequencing reads from the two small RNA libraries were cleaned to remove contaminants such as 5′-primer residues, poly(A) tails, and low-quality reads. Clean reads were then filtered by mapping to the annotated genes of the *Helianthus tuberosus* reference genome, version 4, and the Rfam database to eliminate reads corresponding to protein-coding genes, RNA, tRNA, snRNA, and snoRNA [33]. The remaining reads were used for miRNA identification. Mature miRNA sequences from various plant species were obtained from miRBase 22.1 (https://www.mirbase.org/, accessed on 1 June 2024) and PmiREN 2.0 (https://www.pmiren.com/, accessed on 1 June 2024). After removing duplicates, these sequences served as queries for searching the two sRNA libraries using ncbi-blast-2.14.0 software (https://blast.ncbi.nlm.nih.gov/Blast.cgi, accessed on 10 June 2024) [68]. Sequences with up to three mismatches in the sRNA libraries were considered potential miRNA candidates and were aligned with spliced transcripts of the Jerusalem artichoke transcriptome (cv. NY1, data unpublished) to identify precursor candidates with zero mismatch. Repeats and protein-coding transcripts annotated by NCBI Blastx were excluded. Upstream and downstream sequences of miRNA candidates were extracted from the transcripts: 4000 nt for miR858 and 300 nt for others [32]. These sequences were folded using RNA Folding Form V2.3 (https://www.unafold.org/mfold/applications/rna-folding-form-v2.php, accessed on 20 June 2024) with default parameters [69]. Sequences that formed stem-loop structures with miRNA candidates in the stem-arms were considered authentic precursors, according to established plant miRNA identification criteria [31]. To assess miRNA expression, identified miRNAs were normalized to transcripts per million (TPM).

### 4.4. Target Identification and Other Bioinformatics Analyses

The target genes of miRNAs were predicted using the spliced transcripts of the Jerusalem artichoke transcriptome (cv. NY1) obtained in the previous analysis, utilizing psRNATarget (https://www.zhaolab.org/psRNATarget/, accessed on 20 June 2024). Degradome data analysis was performed using modified methods based on Addo-Quaye et al. and Zhou et al. [34,35]. Three degradome libraries of Jerusalem artichoke (cv. NY1) were analyzed: DEG1 derived from RNA extracted from mixed roots and leaves of seedlings, DEG2 from tubers, and DEG3 from mature leaves during flowering.

MATLAB R2023a programs developed by our team were utilized to locate miRNAs on the Jerusalem artichoke genome, extract precursor sequences, and obtain their 2 kb upstream regions as promoter sequences. *Cis*-acting element prediction in the promoter region was performed using the PlantCARE database. Target transcripts were compared to annotated genes in the reference genome using ncbi-blast-2.14.0 software to identify homologous genes. Open reading frames (ORFs) of genes were predicted using the ORFfinder tool (https://www.ncbi.nlm.nih.gov/orffinder/, accessed on 10 July 2024). Protein physicochemical properties and hydrophilicity were analyzed using the Expasy-ProtParam (https://web.expasy.org/protparam/, accessed on 10 July 2024), and subcellular localization was predicted using Wolfpsort (https://wolfpsort.hgc.jp, accessed on 10 July 2024). Sequence conservation analysis was conducted using WebLogo, revision 3 (https://weblogo.threeplusone.com/create.cgi, accessed on 10 July 2024). Multiple sequence alignment and phylogenetic tree construction were performed using MEGA 7.

### 4.5. cDNA Synthesis and Gene Cloning and Expression

High-quality RNA extracted previously was first reverse-transcribed into cDNA. For sRNA, RNA was reverse-transcribed into cDNA using the PrimeScript™ II 1st Strand cDNA Synthesis Kit (6210A, Takara, Kusatsu, Japan). For other genes, cDNA was synthesized using the HiScript II Q RT SuperMix for qPCR (+gDNA wiper) (R223-01, Vazyme, Nanjing, China), following the manufacturer’s instructions.

For gene cloning, sequences were amplified by PCR using 2 × Hieff^®^ Robust PCR Master Mix (10106ES03, Yeasen, Shanghai, China). PCR products were then isolated from agarose gel using a gel recovery kit (TSP601, Tsingke, Beijing, China), ligated into the pMD-19T vector (6013, Takara, Kusatsu, Japan), transformed into DH5α Chemically Competent Cells (TSC-C14, Tsingke, Beijing, China), and sequenced by Tsingke (Beijing, China).

Quantitative real-time PCR (qRT-PCR) was performed using a Quantagene q225 PCR instrument with ChamQ Universal SYBR qPCR Master Mix (Q711, Vazyme, Nanjing, China) and gene-specific primers. Degenerate primers were used due to the high conservation among MIRNAs/miRNAs of the same family (see Table S8). Primers were designed with Primer-BLAST (https://blast.ncbi.nlm.nih.gov/Blast.cgi, accessed on 15 July 2024) and synthesized by Tsingke (Beijing, China). For sRNA, the qRT-PCR reaction mixture included 2 μL of 5 × diluted cDNA, 2 μL of forward primer, 2 μL of reverse primer, and 4 μL of TB Green Premix Ex Taq, as described by Tang et al. [70]. For other genes, the qRT-PCR reaction mixture consisted of 1 μL of 5 × diluted cDNA, 0.4 μL of forward primer, 0.4 μL of reverse primer, 5 μL of TB Green Premix Ex Taq, and 3.2 μL of sterile water, according to the manufacturer’s instructions. The qRT-PCR procedure involved an initial denaturation step at 95 °C for 30 s, followed by 40 cycles of 5 s at 95 °C and 30 s at 60 °C, as per the manufacturer’s instructions. *HtU6* snRNA and *HtActin* were used as internal controls for sRNA and other genes, respectively. Each gene was assessed with three biological replicates and nine technical replicates. The relative expression levels of miRNAs, their precursors, and targets were calculated using the 2^−ΔΔCt^ method for bar graphs and the −ΔΔCt method for heat maps [71].

### 4.6. Statistical Analysis

All experiments described in this study were conducted with three biological replicates. Statistical analysis was performed using one-way analysis of variance (ANOVA) followed by Duncan’s multiple range test (*p* < 0.05) with Microsoft Excel 2021 and IBM SPSS Statistics 25. Graphs and charts were generated using Adobe Illustrator 2020, GraphPad Prism 8, and TBtools v2.142 [72].

## 5. Conclusions

This study provides a comprehensive analysis of miRNA responses to Cu stress in Jerusalem artichoke. The identification of miR397, miR398, and miR408, along with other conserved miRNAs such as miR168, miR394, and miR858, highlights their pivotal roles in regulating Cu stress responses. Specifically, miR397, miR398, and miR408 were found to be downregulated under Cu stress, which correlates with their regulation of key Cu-containing proteins. Conversely, miR394 and miR858 displayed differential expression patterns that suggest a complex role in tuber development and stress adaptation. This study not only confirms the conserved nature of miRNA regulatory mechanisms across plant species but also provides novel insights into their specific functions in Jerusalem artichoke. These findings contribute to a deeper understanding of miRNA-mediated stress responses and offer potential targets for improving plant stress tolerance through genetic or biotechnological approaches. Future research should further investigate the interactions between these miRNAs and their target genes to elucidate their precise roles in stress adaptation.

## Figures and Tables

**Table 1:** Sequences and differential expression profiles of six miRNA families under copper stress in *Helianthus tuberosus* from sRNA sequencing. Fold change (FC) = Cu stress (TPM)/control (TPM).

miRNA Member	Sequence	Length (nt)	Control (TPM)	Cu Stress (TPM)	Log_2_FC
htu-miR168a	UCGCUUGGUACAGGUCGGGAA	21	1379	1402	0.02
htu-miR168b	UCGCUUGGUGCAGGUCGGGAA	21	1385	3907	1.50
htu-miR394a	UUGGCAUUCUGUCCACCUCC	20	15	5	−1.58
htu-miR397a	UCAUUGAGUGCAGCGUUGAUG	21	112	5	−4.49
htu-miR398a	CGUGUUCUCAGGUCGCCCCUG	21	473	11	−5.43
htu-miR398b	UGUGUUCUCAGGUCGCCCCUG	21	5671	147	−5.27
htu-miR398c	UGUGUUCUCAGGUCACCCCUU	21	5	8	0.68
htu-miR408a	UGCACUGCCUCUUCCCUGGC	20	45	19	−1.24
htu-miR408b	UGCACUGUCUCUUCCCUGGC	20	6	1	−2.58
htu-miR408c	UGCACUGUCUCUUCCCUGUCU	21	16	1	−4.00
htu-miR858a	UUCGUUGUCUGUUCGACCUUG	21	11,740	4480	−1.39

## Data Availability

Data are contained within the article and Supplementary Materials.

## Supplementary Materials

The following supporting information can be downloaded at https://www.mdpi.com/article/10.3390/plants14060955/s1: Figure S1: Venn diagrams illustrating unique (A) and total (B) reads identified in the control and Cu stress libraries from *Helianthus tuberosus*; Figure S2: Distribution of unique (A) and total (B) read categories in the two *Helianthus tuberosus* libraries; Figure S3: Predicted stem-loop structures of MIR168, MIR394, MIR397, MIR398, and MIR408 in *Helianthus tuberosus*; Figure S4: Multiple sequence alignment of six miRNA family members across various plant species; Figure S5: Alignment of the precursors of miR398 (A) and miR408 (B) in *Helianthus tuberosus*; Figure S6: Phylogenetic trees of the precursors of miR168, miR394, miR398, and miR858 in *Helianthus tuberosus*; Figure S7: The predicted *cis*-acting elements in the promoters of *MIRNA* genes in *Helianthus tuberosus*; Figure S8: t-plots of the targets of the miR168, miR394, miR397, miR398, and miR408 identified from three degradome libraries in *Helianthus tuberosus*; Figure S9: Multiple sequence alignment of fifteen *HtMyb* genes targeted by htu-miR858, showing 50 bases from the 9th position of the miR858 binding site (5′→3′); Table S1: Length distribution of unique and total reads in two libraries of *Helianthus tuberosus*; Table S2: Sequence of miRNA* corresponding to six Cu-responsive miRNA families in *Helianthus tuberosus*; Table S3: Genomic localization of six miRNA family members; Table S4: Mature miR858 sequences from various plant species; Table S5: *Cis*-acting elements in the *MIRNA* genes promoters; Table S6: Genome-annotated genes that are highly homologous to the miRNA target genes identified from the Jerusalem artichoke NY1 transcript; Table S7: Property analysis of targets from six miRNA families in *Helianthus tuberosus*; Table S8: The primers used in this study.

## Abbreviations

The following abbreviations are used in this manuscript:

Cu	Copper
miRNA	microRNA
qRT-PCR	quantitative real-time PCR
AGO	Argonaute
LAC	Laccase
CCS	Copper chaperone for superoxide dismutase
CSD	Copper/Zinc superoxide dismutase
BBP	Basic blue protein
MYB	Myeloblastosis
RISC	RNA-induced silencing complex
ORF	Open reading frame
AA	Amino acids
MW	Molecular weight
pI	Isoelectric point
GRAVY	Grand average of hydropathicity
nt	nucleotide
