# Supplementary material for: Identification and Characterization of Copper-Responsive miRNAs and Their Target Genes in Jerusalem Artichoke
Source: Plants (Basel). 2025 Mar 18;14(6):955. doi: 10.3390/plants14060955 (PMC11945104; doi:10.3390/plants14060955)
Supplement: Supplementary file 1 [file plants-14-00955-s001.zip › Figures S1–9 and Tables S1,2,7.pdf]

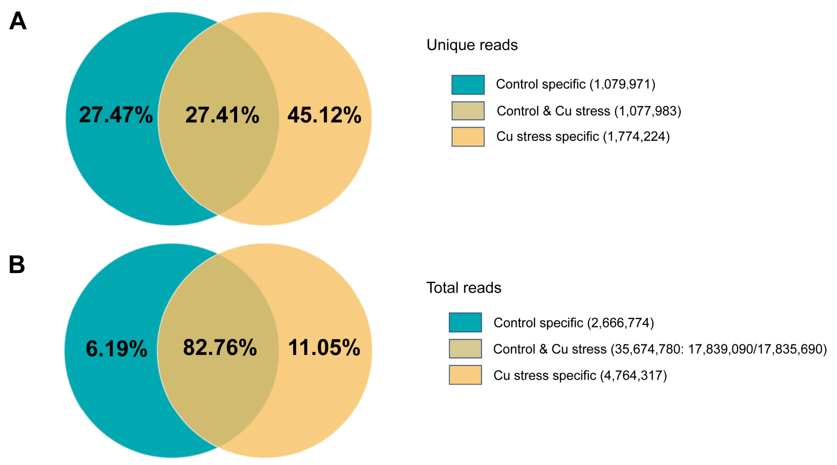

Figure S1. Venn diagrams illustrating unique (A) and total (B) reads identified in the control and Cu stress libraries from *Helianthus tuberosus*.

Percentages and numbers in parentheses indicate the proportion and the specific or shared quantities of reads in the two libraries, respectively.

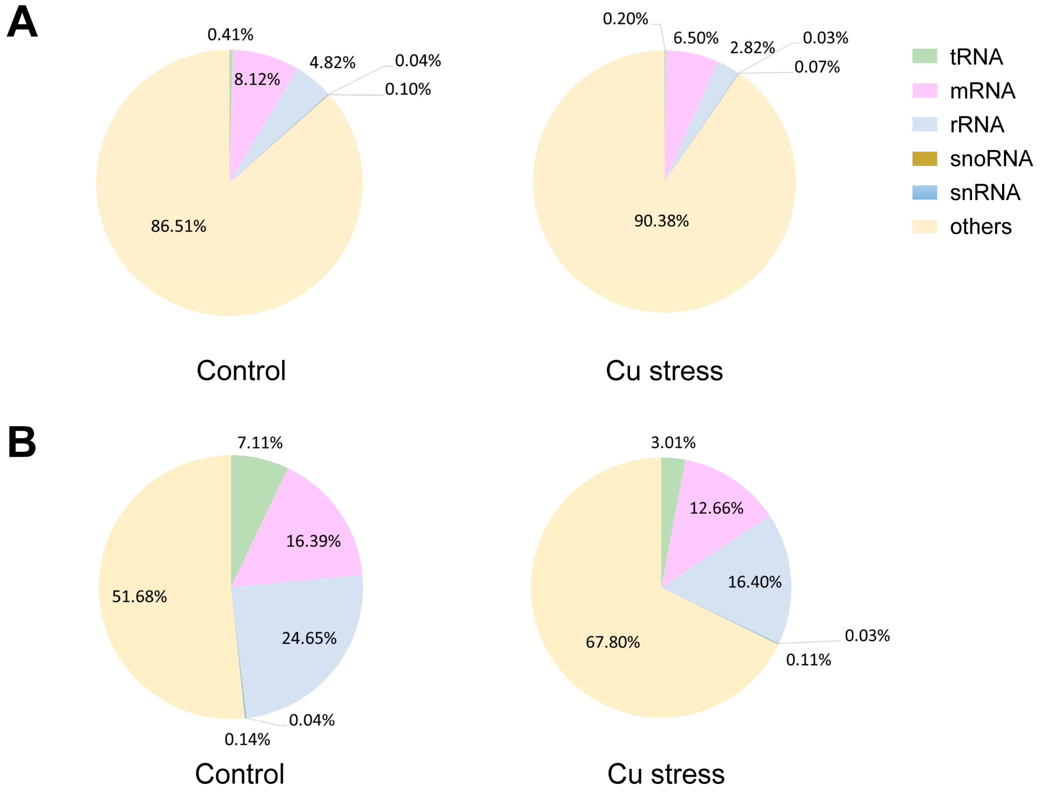

Figure S2. Distribution of unique (A) and total (B) read categories in the two *Helianthus tuberosus* libraries.

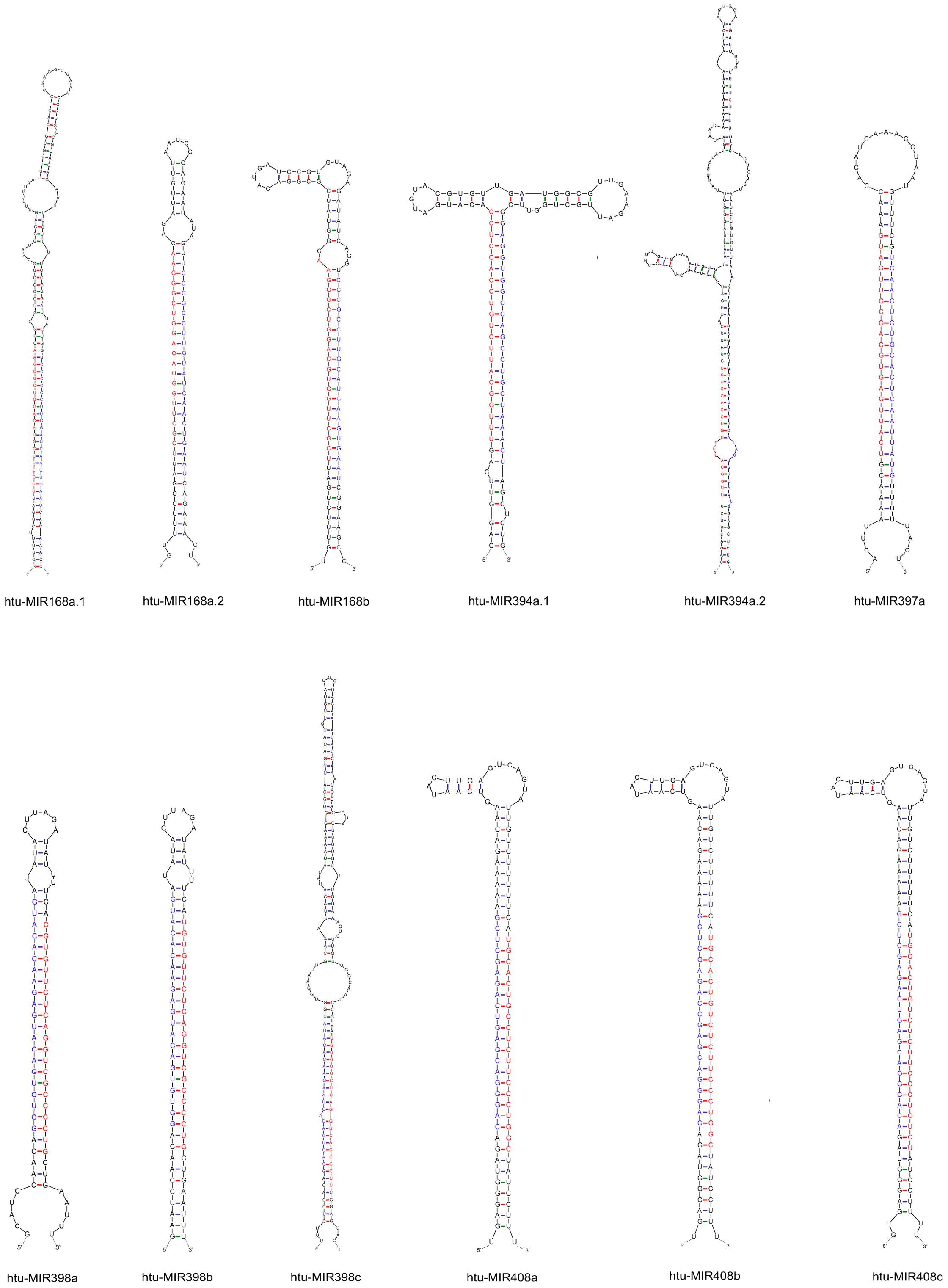

Figure S3. Predicted stem-loop structures of MIR168, MIR394, MIR397, MIR398, and MIR408 in *Helianthus tuberosus*.

The sequences of miRNA and miRNA* are marked in red and blue, respectively.

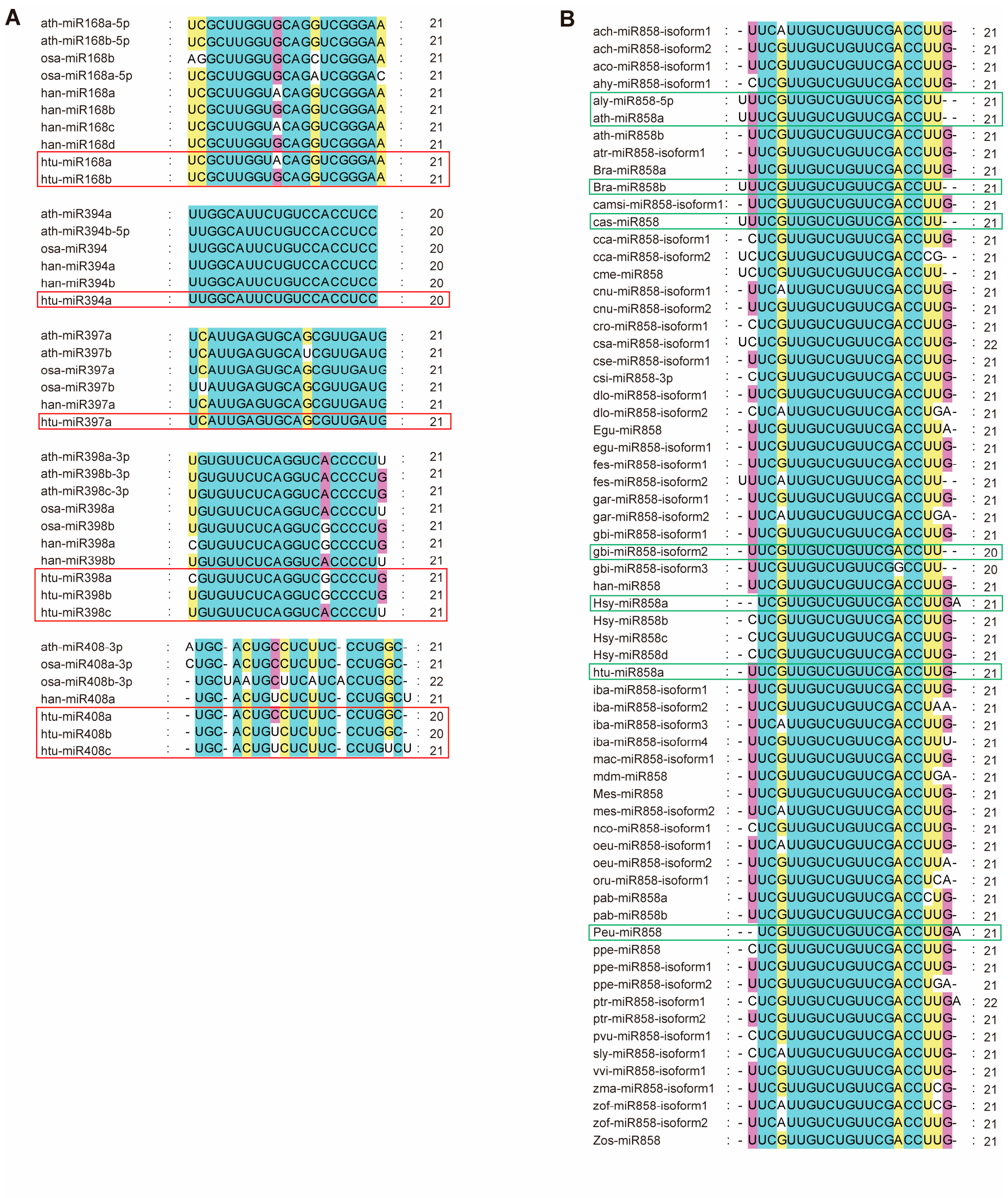

Figure S4. Multiple sequence alignment of six miRNA family members across various plant species. (A) Alignment of miR168, miR394, miR397, miR398, and miR408 from *Arabidopsis thaliana* (Ath), *Oryza sativa* (Osa), *Helianthus annuus* (Han), and *Helianthus tuberosus* (Htu). (B) Alignment of miR858 from various plant species retrieved from miRBase, PmiREN, and Wang et al. (2023) (see Supplementary Table 3). The red rectangle indicates miRNA sequences, while the green box highlights unique miR858 isoforms identified in *Helianthus tuberosus* in this study. Alignment was conducted using MEGA7 and visualized with GENEDOC.

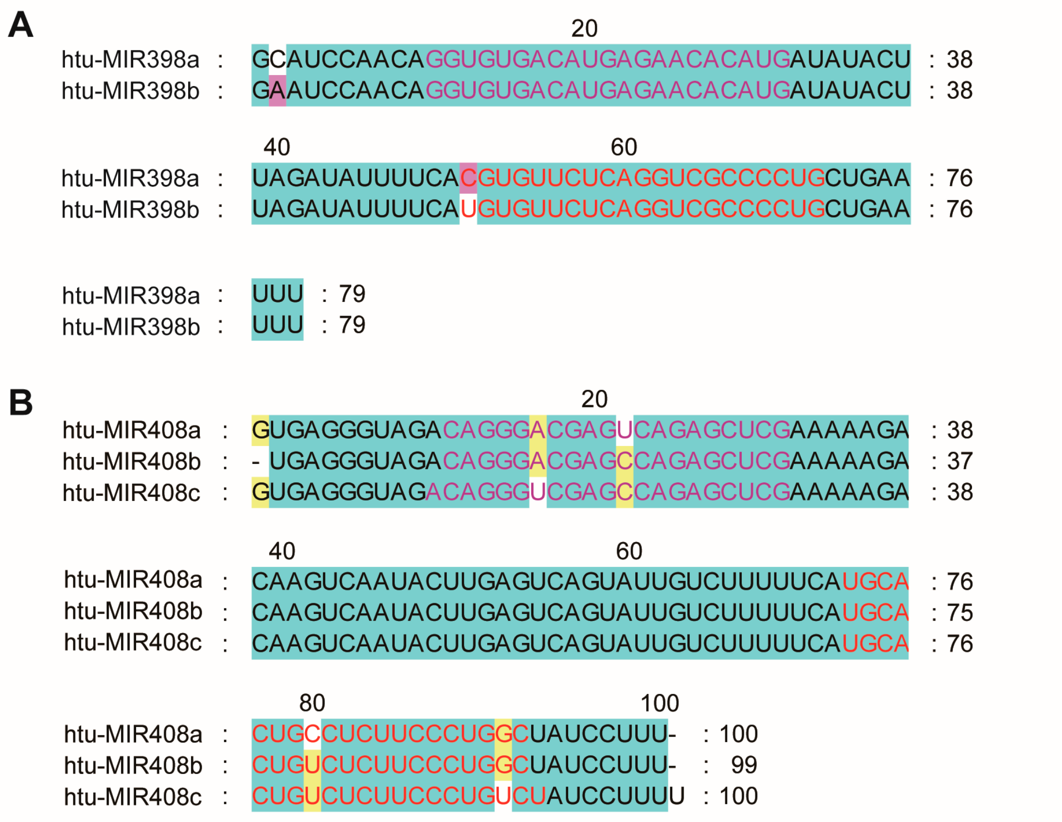

Figure S5. Alignment of the precursors of miR398 (A) and miR408 (B) in *Helianthus tuberosus*. The sequences of miRNA and miRNA* are highlighted in red and purple, respectively.

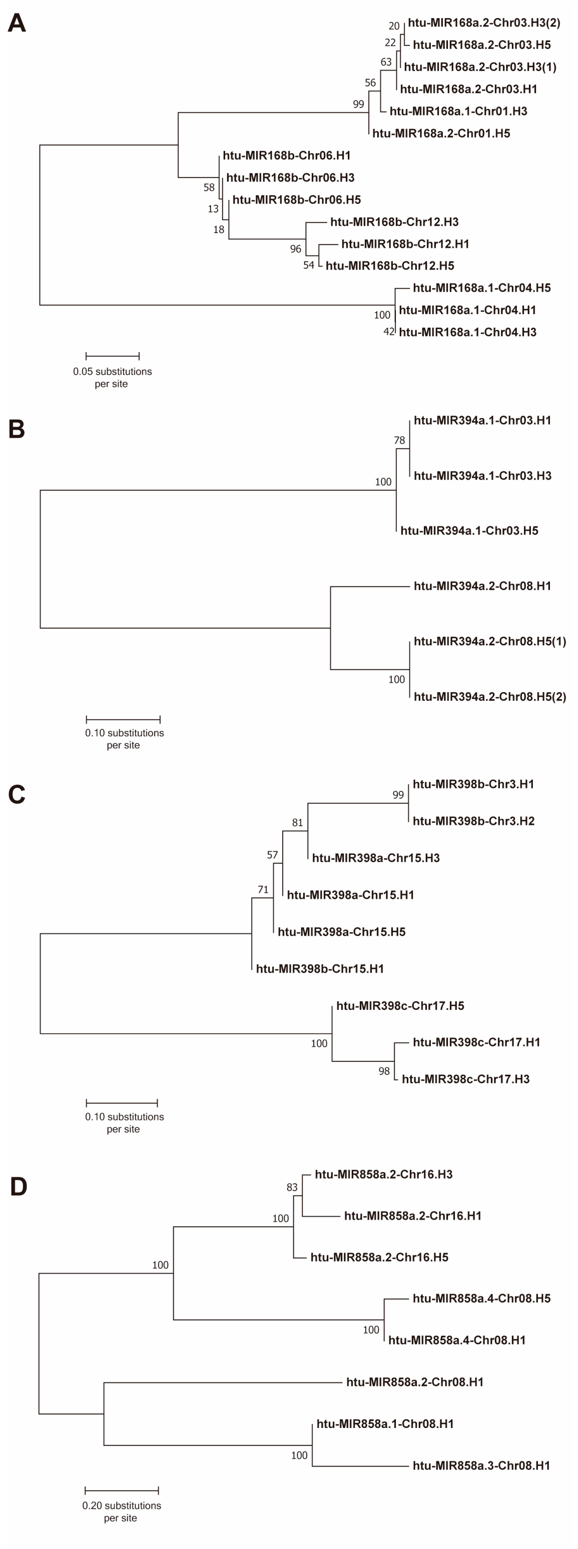

Figure S6. Phylogenetic trees of the precursors of miR168, miR394, miR398, and miR858 in *Helianthus tuberosus*.

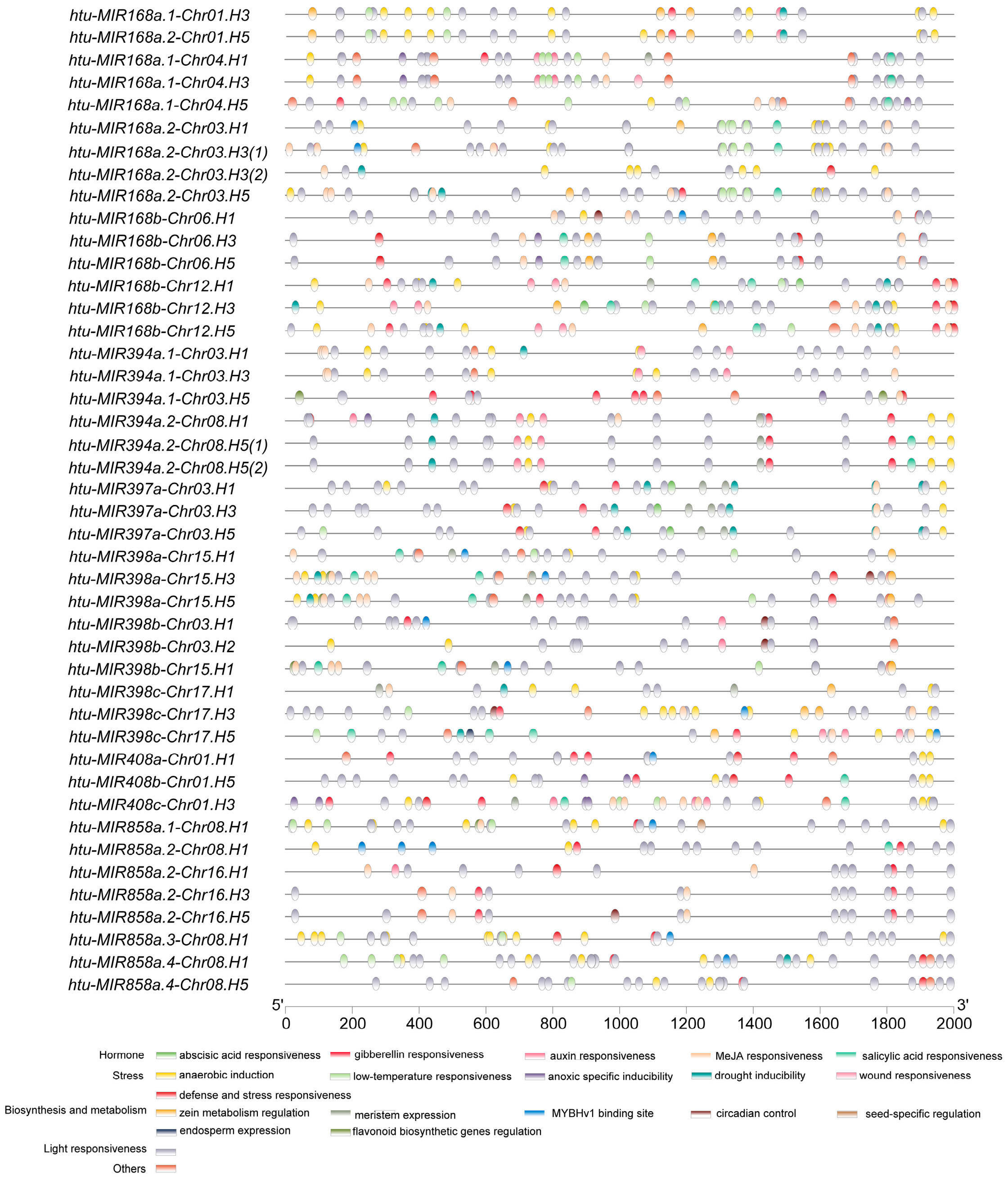

Figure S7. The predicted *cis*-acting elements in the promoters of *MIRNA* genes in *Helianthus tuberosus*.

The 2.0 kb upstream regions of the *MIRNA* genes were analyzed and predicted using the PlantCARE database and subsequently visualized using TBtools.

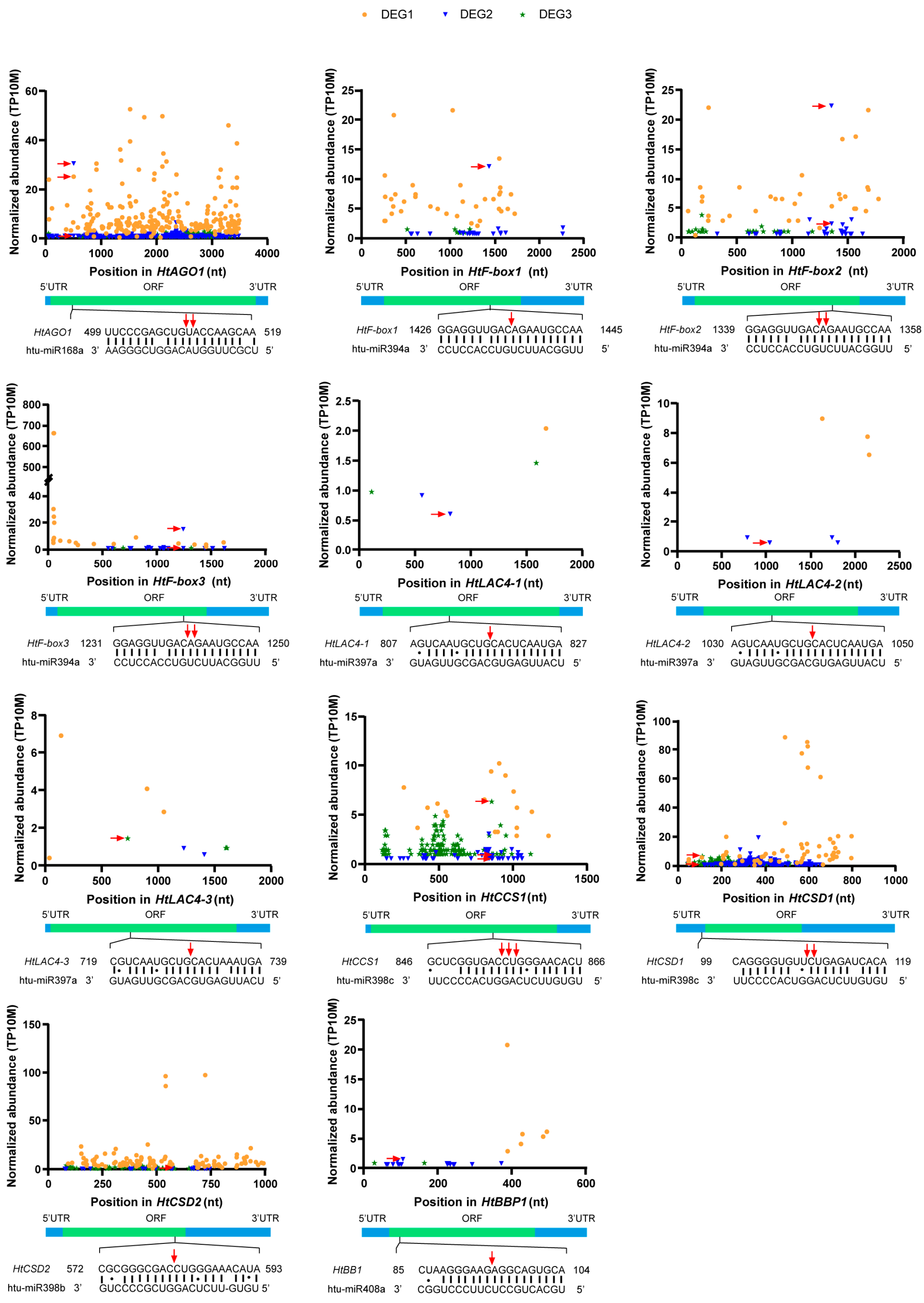

Figure S8. t-plots of the targets of the miR168, miR394, miR397, miR398, and miR408 identified from three degradome libraries in *Helianthus tuberosus*.

Eleven genes were targeted by miRNA. Refer to Fig. 5 for additional information.

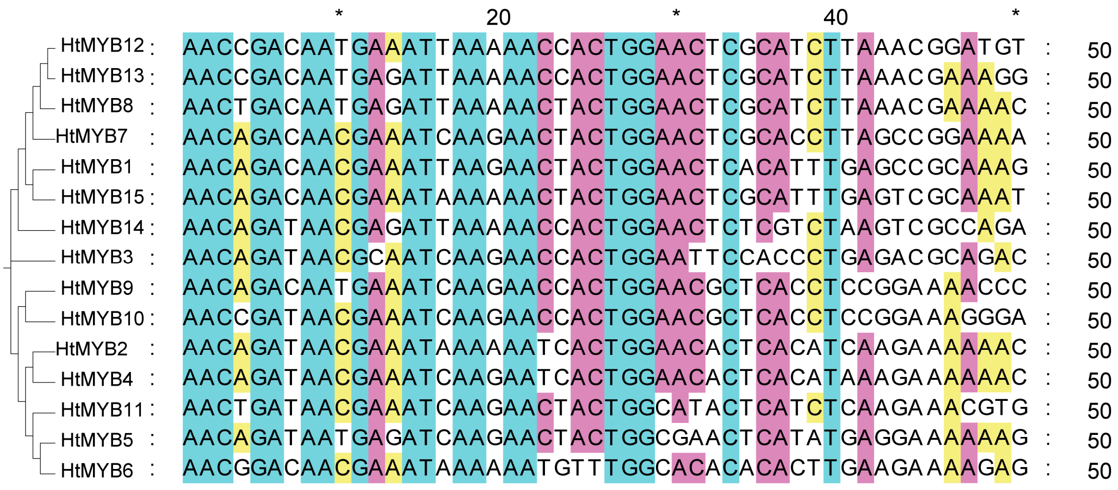

Figure S9. Multiple sequence alignment of fifteen *HtMyb* genes targeted by htu-miR858, showing 50 bases from the 9th position of the miR858 binding site (5′→3′).

Table S1. Length distribution of unique and total reads in two libraries of *Helianthus tuberosus*.

Length (nt)	CK-	%	CK-total	%	Cu-unique	%	Cu-total	%
All	2157954	100.0	20505864	100.0	2852207	100.0	22600007	100.00
19	31936	1.48	636282	3.10	38071	1.33	572722	2.53
20	44434	2.06	658008	3.21	58785	2.06	696100	3.08
21	246064	11.40	5261541	25.66	321408	11.27	6182004	27.35
22	149618	6.93	1603850	7.82	208132	7.30	1954872	8.65
23	279535	12.95	1368756	6.67	429327	15.05	1958217	8.66
24	1188635	55.08	5113354	24.94	1603275	56.21	7538708	33.36
25	33809	1.57	338451	1.65	51327	1.80	351702	1.56
Sum	1974031	91.48	14980242	73.05	2710325	95.03	19254325	85.20

Table S2. Sequence of miRNA* corresponding to six Cu-responsive miRNA families in *Helianthus tuberosus*.

miRNA member	Sequence	Length (nt)	Control reads	Cu-stress reads
htu-miR168a.1*	CCCGCCTTGGATCAACTGAAT	21	104	78
htu-miR168a.2*	CCCGCCTTGTATCAACTGAAT	21	270	153
htu-miR168b*	CCCGCCTTGCATCAAGTGAAT	21	567	622
htu-miR394a.1*	AGGTGGCCAGCCTGCTAAACT	21	5	0
htu-miR394a.2*	AGGTGGGCATACTGTCAATG	20	22	32
htu-miR397a*	TCAACTCTGCACTCAATTATG	21	2132	406
htu-miR398a*	GGTGTGACATGAGAACACATG	21	74	8
htu-miR398b*	GGTGTGACATGAGAACACATG	21	74	8
htu-miR398c*	GGAGTGACACTAAGAACACATA	22	0	5
htu-miR408a*	CAGGGACGAGTCAGAGCTCG	20	2	0
htu-miR408b*	CAGGGACGAGCCAGAGCTCG	20	0	0
htu-miR408c*	ACAGGGTCGAGCCAGAGCTCG	21	69	0
htu-miR858a.1*	AGGTGGAACAGTCAATGAATG	21	0	0
htu-miR858a.2*	AGGTGGAACAGACGATGAATG	21	12	0
htu-miR858a.3*	AGGTGGAACAGACAATGAATG	21	0	0
htu-miR858a.4*	AGGTGGGACAGACAATGAATG	21	1	0

Table S7. Properties analysis of targets from six miRNA families in *Helianthus tuberosus*. AA: Amino acids; MW: Molecular weight; pI: Isoelectric point; GRAVY: Grand average of hydropathicity.

NO	Gene name	Accession number	miRNA	Category of targets	CDS length (bp)	Number of AA	MW (kDa)	pI	GRAVY	Subcellular localization
1	*HtAGO1*	PQ368255	miR168	I	3177	1058	117.49	9.43	−0.571	nucleus
2	*HtF-box1*	PQ368256	miR394	I	1320	439	49.63	9.05	−0.052	cytoplast
3	*HtF-box2*	PQ368257	miR394	I	1401	466	53.41	9.04	−0.244	peroxisome
4	*HtF-box3*	PQ368258	miR394	I	1359	452	51.05	8.58	−0.210	endoplasmic reticulum
5	*HtLAC4-1*	PQ368259	miR397	II	1680	559	61.30	9.28	0.010	chloroplast
6	*HtLAC4-2*	PQ368260	miR397	II	1689	562	61.72	9.21	0.044	chloroplast
7	*HtLAC4-3*	PQ368261	miR397	I	1668	555	60.81	9.33	−0.040	chloroplast
8	*HtCCS1*	PQ368262	miR398	I	948	315	33.44	6.14	−0.100	chloroplast
9	*HtCSD1*	PQ368263	miR398	I	462	153	15.43	5.61	−0.154	cytoplast
10	*HtCSD2*	PQ368264	miR398	III	534	177	18.25	6.33	−0.063	chloroplast
11	*HtBBP1*	PQ368265	miR408	I	381	126	13.12	9.68	0.183	extracellular
12	*HtMYB1*	PQ368266	miR858	I	723	240	27.22	5.45	−0.875	nucleus
13	*HtMYB2*	PQ368267	miR858	I	822	273	30.94	5.03	−0.736	nucleus
14	*HtMYB3*	PQ368268	miR858	I	957	318	34.46	5.66	−0.453	nucleus
15	*HtMYB4*	PQ368269	miR858	I	939	312	35.32	4.84	−0.579	nucleus
16	*HtMYB5*	PQ368270	miR858	I	765	254	29.33	8.96	−0.912	nucleus
17	*HtMYB6*	PQ368271	miR858	I	819	272	31.25	6.20	−0.797	nucleus

NO	Gene name	Accession number	miRNA	Category of targets	CDS length (bp)	Number of AA	MW (kDa)	pI	GRAVY	Subcellular localization
18	*HtMYB7*	PQ368272	miR858	I	912	303	33.73	5.39	−0.686	nucleus
19	*HtMYB8*	PQ368273	miR858	I	762	253	86.71	4.99	0.832	nucleus
20	*HtMYB9*	PQ368274	miR858	I	768	255	95.75	5.07	0.783	nucleus
21	*HtMYB10*	PQ368275	miR858	I	783	260	84.01	5.10	0.770	nucleus
22	*HtMYB11*	PQ368276	miR858	I	750	249	163.63	4.92	0.731	nucleus
23	*HtMYB12*	PQ368277	miR858	I	744	247	93.87	5.07	0.772	nucleus
24	*HtMYB13*	PQ368278	miR858	I	768	255	92.51	5.06	0.800	nucleus
25	*HtMYB14*	PQ368279	miR858	I	948	315	139.70	5.06	0.650	nucleus
26	*HtMYB15*	PQ368280	miR858	I	852	283	106.18	5.05	0.759	nucleus
